# Asymmetric Epoxidation of Olefins with Sodium Percarbonate Catalyzed by *Bis*-amino-*bis*-pyridine Manganese Complexes

**DOI:** 10.3390/molecules27082538

**Published:** 2022-04-14

**Authors:** Varvara A. Drozd, Roman V. Ottenbacher, Konstantin P. Bryliakov

**Affiliations:** Boreskov Institute of Catalysis, Pr. Lavrentieva 5, 630090 Novosibirsk, Russia; v.drozd@g.nsu.ru

**Keywords:** enantioselective, epoxidation, manganese, sodium percarbonate, olefin, alkene

## Abstract

Asymmetric epoxidation of a series of olefinic substrates with sodium percarbonate oxidant in the presence of homogeneous catalysts based on Mn complexes with bis-amino-bis-pyridine ligands is reported. Sodium percarbonate is a readily available and environmentally benign oxidant that is studied in these reactions for the first time. The epoxidation proceeded with good to high yields (up to 100%) and high enantioselectivities (up to 99% *ee*) using as low as 0.2 mol. % catalyst loadings. The epoxidation protocol is suitable for various types of substrates, including unfunctionalized alkenes, *α*,*β*-unsaturated ketones, esters (*cis*- and *trans*-), and amides (*cis*- and *trans*-). The reaction mechanism is discussed.

## 1. Introduction

Chiral epoxides are useful building blocks in organic synthesis and essential synthetic targets [1,2,3]. The demand for synthetic methodologies of chiral epoxides preparation has been nourished by the biological activities exhibited by various natural products containing an epoxide unit and their applications as convenient (stable yet readily reactive) precursors to more complex chiral molecules [4,5,6]. The production of epoxides from the corresponding olefins by asymmetric epoxidation reaction in the presence of transition metal catalysts is considered the most efficient and versatile method [7,8,9,10,11]. In this realm, manganese(II) complexes with chiral *N*_4_ *bis*-amino-*bis*-pyridine and related ligands were established as highly enantioselective and efficient catalysts of olefins epoxidation with the environmentally benign oxidant hydrogen peroxide [12,13,14,15]. In the recent decade, the topic has been extensively studied by groups of Sun [16,17,18,19,20,21,22,23], Costas [24,25,26,27], Bryliakov [12,28,29,30,31,32], and others [33,34,35]. Using hydrogen peroxide in these reactions is considered beneficial for several reasons: aqueous H_2_O_2_ is a safe, easy-to-handle oxidant with high active oxygen content (47%), which produces water as the only by-product. Nonetheless, it is known that hydrogen peroxide is prone to disproportionation in solutions containing transition metals like iron or manganese, which may significantly deteriorate the oxidant efficiency. Typically, this is partially sorted out via slow, syringe-pump oxidant addition. Other oxidants, including peracids, alkylhydroperoxides, and iodosylarenes, have also been utilized in *bis*-amino-*bis*-pyridine manganese complexes catalyzed epoxidation [31,36]. We present the use of sodium percarbonate as a convenient and environmentally benign solid oxidant for manganese catalyzed enantioselective epoxidation, which is added to the reaction mixture in portions. The corresponding epoxides of various olefins were obtained in good to quantitative yields with up to 99% *ee*.

## 2. Results and Discussion

**Figure 1:**
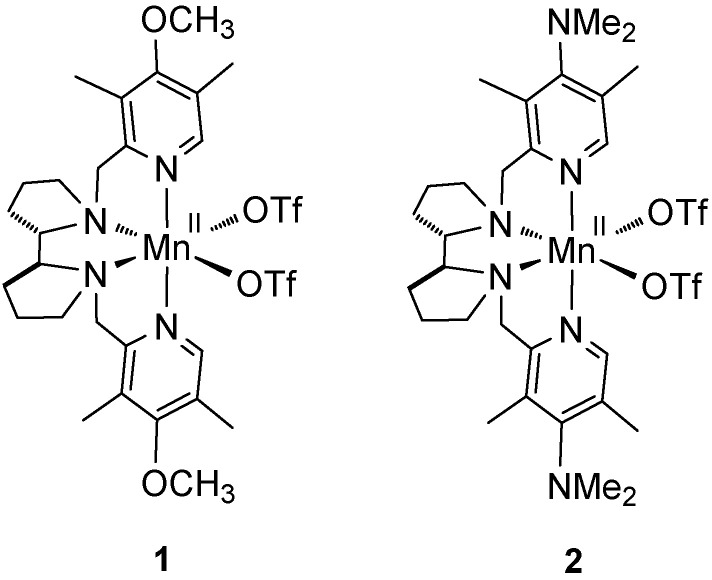
Manganese complexes used in this study. OTf = trifluoromethanesulfonate.

The commercial bleaching agent sodium percarbonate (Na_2_CO_3_ 1.5H_2_O_2_) is a white powder stable at room temperature [37]. It has no shock sensitivity and contains 15% of active oxygen. Previously, sodium percarbonate was utilized in various oxidation reactions, including oxidations of sulfides to sulfones, anilines to nitroarenes, and non-enantioselective epoxidations [37,38]. In order to find appropriate conditions for employing sodium percarbonate in manganese-catalyzed asymmetric epoxidation, we initially tested it in reaction with chalcone in the presence of catalyst **1** [30] (Figure 1).

The epoxidations with H_2_O_2_ in the presence of *bis*-amino-*bis*-pyridine manganese complexes usually require adding carboxylic acid as a co-catalytic additive [28,29]. Herewith, using acetic acid, AcOH, as an additive (14 equiv. vs. chalcone) and sodium percarbonate (2 equiv. vs. chalcone, added in one portion) as an oxidant resulted in a nearly quantitative formation of chalcone epoxide having 82% *ee* (Table 1, entry 1). To improve the enantioselectivity of the reaction, a more sterically demanding 2-ethylbuthanoic acid (EBA) [20,29] was probed (Table 1, entry 2). Indeed, the enantioselectivity increased up to 94% *ee*, albeit with a reduced conversion of 83%. Raising the amount of oxidant to 2.5 equiv. vs. substrate led to only a minor increase in epoxide yield (92%, Table 1, entry 3). Adding sodium percarbonate in three portions within 30 min intervals was revealed as the most practical protocol, furnishing nearly quantitative conversion of chalcone to the epoxide having 94% *ee* (Table 1, entry 4).

**Figure 2:**
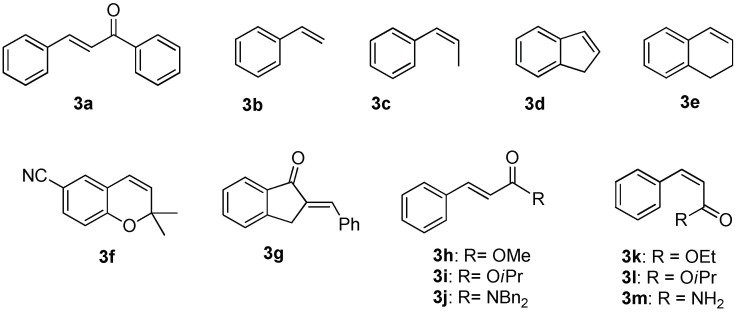
Olefinic substrates studied in manganese catalyzed epoxidation with sodium percarbonate.

Having these optimized conditions in hand, we carried out the asymmetric epoxidation of a series of substrates (Figure 2) in the presence of Mn complex **1** (Table 2). The epoxidation of unfunctionalized alkenes **3b**–**e** (Table 2, entries 1–4) afforded the corresponding epoxides with high yields (95–100%) and moderate to good enantioselectivity (51–79% *ee*). The epoxidation of 2,2-dimethyl-2*H*-chromene-6-carbonitrile **3f** to the corresponding epoxide (a precursor for the antihypertensive agent *levcromakalim* [39]) was accomplished in 99% yield and 95% *ee* (Table 2, entry 5). Substrate **3g**, bearing *α,β*-unsaturated ketone functionality, was epoxidized with moderate conversion under these conditions (47% yield, Table 2, entry 6). Nonetheless, the enantioselectivity was high (87% *ee*). The epoxidation of *trans*-*α,β*-unsaturated esters **3h** and **3i** demonstrated the dependence of asymmetric induction on the steric demand of alkyl substituents in the ester group (*cf*. 87% *ee* for –O*i*Pr vs. 80% *ee* for –OMe, Table 2, entries 7,8), in full accordance with previous observations [30]. Highly enantioselective epoxidation (99% *ee*) of *trans*-enamide **3j** was documented (Table 2, entry 9), although it required increased catalyst loading of 0.5 mol. % and was accomplished in moderate yield (60%). The same amount of the catalyst was enough to mediate the asymmetric epoxidation of *cis*-enamide **3m** with 81% yield and 79% *ee* (Table 2, entry 12). The esters of *cis*-cinnamic acid **3k** and **3l** were converted to corresponding epoxides with high yields (100 and 96%, respectively); the enantioselectivity was higher for the bulkier –O*i*Pr ester (94% *ee*, Table 2, entry 11), cf. 86% *ee* for the–OEt ester (Table 2, entry 10).

Based on earlier data [32], one could expect that increasing the electron-donating ability of the ligands of the Mn-based catalysts should enhance the epoxidation enantioselectivity. Indeed, catalyst **2 [30]**, bearing stronger electron-donating NMe_2_ groups at the pyridylmethyl moieties of the ligand (Figure 1), in all cases but **3j**, showed higher enantioselectivities (Table 3), which improvement was most significant in the case of unfunctionalized alkenes **3b**–**e** (Table 3, entries 2–5). For the epoxidation of *trans*-*α,β*-unsaturated esters **3h** and **3i**, the steric hindrance did not affect the asymmetric induction (86 and 87% *ee*, respectively, Table 3, entries 8, 9; cf. entries 6, 7 of Table 2). *cis*-Cinnamic acid derivatives **3k**-**m** were epoxidized in high yields (84–98%) and enantioselectivities (82–95% *ee*, Table 3, entries 11–13). Olefins **3a**, **3f,** and **3j** were converted to the corresponding epoxides almost quantitatively, with excellent enantioselectivity (95–97% *ee*, Table 3, entries 1, 6, 10).

It was reported previously [38] that sodium percarbonate is prone to deliver hydrogen peroxide in the reaction medium. The intermediate formation of peroxycarboxylic acid can be ruled out as far as under near-anhydrous conditions it is possible only from carboxylic acid anhydrides or chloroanhydrides rather than the acid itself [37]. Therefore, one can suggest that the slowly liberated H_2_O_2_ acts as a true oxidant. We have established that for *bis*-amino-*bis*-pyridine manganese complexes-catalyzed asymmetric epoxidation with H_2_O_2_, the addition of carboxylic acid is required to achieve reasonable conversions [29,30]. The latter is assumed to promote the heterolytic cleavage of the O-O bond in the (L)Mn^III^OOH intermediate to generate the (L)Mn^V^ = O active species, responsible for the enantioselective oxygen transfer [30,31].

## 3. Materials and Methods

### 3.1. Materials

All chemicals and solvents were purchased from Aldrich, Acros Organics, or Alfa Aesar and were used without additional purification unless noted otherwise. For catalytic epoxidation experiments, technical grade sodium percarbonate (Na_2_CO_3_ 1.5H_2_O_2_) was used. Chiral Mn catalysts **1** and **2** were prepared as described [30] and were recrystallized from acetonitrile/diethyl ether. Substrates **3a**–**f** were purchased and used without further purification; others were prepared as described [12,32].

### 3.2. Instrumentation

^1^H NMR spectra were measured on Bruker Avance 400 spectrometer at 400.13 MHz and on Bruker DPX-250 spectrometer at 250.13 MHz, respectively. Chemical shifts were internally referenced to the residual proton signal of CDCl_3_ (7.26 ppm) for ^1^H NMR spectra. The enantiomeric excess values of chiral epoxides were measured by HPLC (Shimadzu LC-20 chromatograph,) equipped with a set of chiral columns (Daicel) as described [12,30,32].

### 3.3. General Procedure for the Catalytic Epoxidation of Olefins with Sodium Percarbonate

In a typical experiment, substrate (100 μmol) and carboxylic acid (1.4 mmol) were added to the solution of the manganese catalyst (0.2 μmol) in CH_3_CN (0.4 mL), and the mixture was thermostated at −40 °C. Then, 200 μmol of mortar-grounded sodium percarbonate was added to the reaction mixture in 3 roughly equal portions, with 30 min intervals between the additions (66.7 μmol in each portion). The resulting mixture was stirred for 2 h at −40 °C (total reaction time: 3 h). The reaction was quenched with a saturated aqueous solution of Na_2_CO_3,_ and the products were extracted with Et_2_O (3 × 4 mL). The solvent was evaporated, and the residue was analyzed by ^1^H NMR spectroscopy (Table S1, Figure S1, SI) to determine conversions and yields and by HPLC on chiral stationary phases (Table S2, Figure S2, SI) to measure the enantiomeric excess values of the chiral epoxides as previously described [12,30,32].

## 4. Conclusions

In conclusion, we have demonstrated that sodium percarbonate can be a convenient oxidant in the asymmetric epoxidation of olefins catalyzed by *bis*-amino-*bis*-pyridine manganese complexes. The epoxidation of various types of substrates, including unfunctionalized alkenes, *α*,*β*-unsaturated ketones, esters (*cis*- and *trans*-), and amides (*cis*- and *trans*-), proceeded with good to high yields (up to 100%) and high enantioselectivities (up to 99% *ee*) using as low as 0.2 mol. % of catalyst loadings. It is assumed that sodium percarbonate releases hydrogen peroxide in the catalytic epoxidation leading to the formation of the reputed manganese(V)-oxo oxygen transferring species. The advantage of the designed epoxidation protocol is the absence of necessity for syringe pump addition of the oxidant. We foresee further studies involving sodium percarbonate oxidant in other manganese catalyzed chemo- and stereoselective oxidations.

## Figures and Tables

**Table 1:** Asymmetric epoxidation of chalcone with sodium percarbonate in the presence of catalyst **1** ^1^.

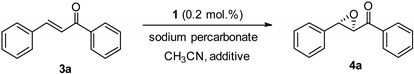				
Entry	Oxidant Equiv.	Additive	Conversion/Yield, %	*ee*, %
1	2.0	AcOH	99/97	82
2	2.0	EBA	83/81	94
3	2.5	EBA	94/92	94
4	2.0 ^2^	EBA	100/97	94

^1^ Reaction conditions: −40 °C, [Mn]/[oxidant]/[chalcone]/[additive] = 0.2 μmol:200 μmol:100 μmol:1.4 mmol in CH_3_CN (0.4 mL), oxidant was added in one portion. ^2^ Oxidant was added in 3 portions within 30 min intervals.

**Table 2:** Asymmetric epoxidation of olefins with sodium percarbonate in the presence of **1** ^1^.

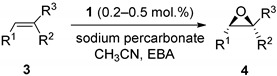				
Entry	Substrate	Cat. Loadings, %	Conversion/Yield, %	*ee*, %
1	**3b**	0.2	100/100	62
2	**3c**	0.2	100/99	79
3	**3d**	0.2	95/95	63
4	**3e**	0.2	98/84	51
5	**3f**	0.2	99/99	95
6 ^2^	**3g**	0.2	47/47	87
7	**3h**	0.2	83/83	80
8	**3i**	0.2	83/83	87
9 ^3^	**3j**	0.5	60/60	99
10	**3k**	0.2	100/100	86
11	**3l**	0.2	96/96	94
12	**3m**	0.5	86/69	79

^1^ Reaction conditions: −40 °C, [Mn]/[oxidant]/[substrate]/[additive] = 0.2 μmol:200 μmol:100 μmol:1.4 mmol in CH_3_CN (0.4 mL), oxidant was added in 3 portions within 30 min intervals. ^2^ Mixed CH_3_CN/CH_2_Cl_2_ (0.4 mL/0.4 mL) solvent was used. ^3^ Mixed CH_3_CN/CH_2_Cl_2_ (0.4 mL/0.6 mL) solvent was used.

**Table 3:** Asymmetric epoxidation of olefins with sodium percarbonate in the presence of **2** ^1^.

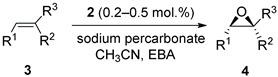				
Entry	Substrate	Cat. Loadings, %	Conversion/Yield, %	*ee*, %
1	**3a**	0.2	99/96	96
2	**3b**	0.2	75/75	67
3	**3c**	0.2	71/71	82
4	**3d**	0.2	100/98	71
5	**3e**	0.2	100/100	60
6	**3f**	0.2	100/100	95
7 ^2^	**3g**	0.2	77/77	82
8	**3h**	0.2	61/61	86
9	**3i**	0.2	46/46	87
10 ^3^	**3j**	0.5	100/100	97
11	**3k**	0.2	98/98	84
12	**3l**	0.2	84/84	95
13	**3m**	0.5	90/90	82

^1^ Reaction conditions: −40 °C, [Mn]/[oxidant]/[substrate]/[additive] = 0.2 μmol:200 μmol:100 μmol:1.4 mmol in CH_3_CN (0.4 mL), oxidant was added in 3 portions within 30 min intervals. ^2^ Mixed CH_3_CN/CH_2_Cl_2_ (0.4 mL/0.4 mL) solvent was used. ^3^ Mixed CH_3_CN/CH_2_Cl_2_ (0.4 mL/0.6 mL) solvent was used.

## Data Availability

Not applicable.

## Supplementary Materials

The following supporting information (SI) can be downloaded at: https://www.mdpi.com/article/10.3390/molecules27082538/s1, Table S1: ^1^H NMR data for the epoxides; Table S2: HPLC data for the epoxides; Figure S1: Selected examples of ^1^H NMR spectra of reaction mixtures; Figure S2: Selected examples of chiral HPLC chromatograms of reaction mixtures.

## Sample Availability

Samples of the compounds used in this paper are available from the authors.
