# Supplementary material for: Asymmetric Epoxidation of Olefins with Sodium Percarbonate Catalyzed by Bis-amino-bis-pyridine Manganese Complexes
Source: Molecules. 2022 Apr 14;27(8):2538. doi: 10.3390/molecules27082538 (PMC9027068; doi:10.3390/molecules27082538)

Electronic Supplementary Information

Asymmetric epoxidation of olefins with sodium percarbonate catalyzed by *bis*-amino-*bis*-pyridine manganese complexes

Varvara A. Drozd [1], Roman V. Ottenbacher [1] and Konstantin P. Bryliakov [1*]

[1] *Boreskov Institute of Catalysis, Pr. Lavrentieva 5, Novosibirsk 630090, Russian Federation*

* *Corresponding author, e-mail: bryliako@catalysis.ru*

Table of contents

Table S1. ^{1}H NMR data for the epoxides. **2**

Table S2. HPLC data for the epoxides. **3**

Figure S1. Selected examples of ^{1}H NMR spectra of reaction mixtures. **4**

Figure S2. Selected examples of chiral HPLC chromatograms of reaction mixtures. **17**

Table S1. ^{1}H NMR data for the epoxides.

Epoxide	^{1}H NMR data
**4a**	δ (250 MHz, $CDCl_3$): 8.04-7.97 (m, 2H), 7.66-7.58 (m, 1H), 7.49 (t, 2H, *J* = 7.9 Hz), 7.42-7.34 (m, 5H), 4.30 (d, 1H, *J* = 1.9 Hz), 4.08 (d, 1H, *J* = 1.9 Hz)
**4b**	δ (400 MHz, $CDCl_3$): 7.38-7.25 (m, 5H), 3.87 (dd, 1H, *J* = 2.6 and 4.0 Hz), 3.15 (dd, 1H, *J* = 4.0 and 5.6 Hz), 2.80 (dd, 1H, *J* = 5.6 and 2.6 Hz)
**4c**	δ (250 MHz, $CDCl_3$): 7.40-7.27 (m, 5H), 4.06 (d, 1H, *J* = 4.2 Hz), 3.34 (dq, 1H, *J* = 4.2 and 5.4 Hz), 1.09 (d, 3H, *J* = 5.5 Hz)
**4d**	δ (250 MHz, $CDCl_3$): 7.51 (d, 1H, *J* = 7.1 Hz), 7.32-7.12 (m, 3H), 4.27 (dd, 1H, *J* = 2.9 and 1.0 Hz), 4.14 (t, 1H, *J* = 2.9 Hz), 3.23 (d, 1H, *J* = 18.0 Hz), 2.99 (dd, 1H, *J* = 18.0 and 2.9 Hz)
**4e**	δ (250 MHz, $CDCl_3$): 7.39 (dd, 1H, *J* = 7.0 and 1.8 Hz), 7.25-7.18 (m, 2H), 7.09 (d, *J* = 7.0 Hz), 3.85 (d, 1H, *J* = 4.2 Hz), 3.73 (t, 1H, *J* = 3.5 Hz), 2.79 (td, 1H, *J* = 14.5 and 6.6 Hz), 5.53 (dd, 1H, *J* = 15.4 and 5.5 Hz), 2.42 (dddd, 1H, *J* = 14.4, 6.5, 2.9 and 1.7 Hz), 1.77 (m, 1H)
**4f**	δ (400 MHz, $CDCl_3$): 7.64 (d, 1H, *J* = 2.0 Hz), 7.51 (dd, 1H, *J* = 8.5 and 2.1 Hz), 6.85 (d, 1H, *J* = 8.5 Hz), 3.90 (d, 1H, *J* = 4.4 Hz), 3.53 (d, 1H, *J* = 4.4 Hz), 1.58 (s, 3H), 1.28 (s, 3H)
**4g**	δ (250 MHz, $CDCl_3$): 7.85 (d, 1H, *J* = 7.6 Hz), 7.71-7.54 (m, 2H), 7.48-7.28 (m, 6H), 4.48 (s, 1H), 3.26 (d, 1H, *J* = 18.3 Hz), 2.93 (d, 1H, *J* = 18.3 Hz)
**4h**	δ (250 MHz, $CDCl_3$): 7.40-7.27 (m, 5H), 4.09 (d, 1H, *J* = 1.7 Hz), 3.82, (s, 3H), 3.51 (d, 1H, *J* = 1.7 Hz)
**4i**	δ (250 MHz, $CDCl_3$): 7.39-7.23 (m, 5H), 5.12 (sept, 1H, *J* = 6.3 Hz), 4.05 (d, 1H, *J* = 1.7 Hz), 3.46 (d, 1H, *J* = 1.7 Hz), 1.30 (d, 3H, *J* = 6.3 Hz), 1.28 (d, 3H, *J* = 6.3 Hz)
**4j**	δ (250 MHz, $CDCl_3$): 7.40-7.10 (m, 15H), 4.65 (d, 2H, *J* = 4.7 Hz), 4.55 (s, 2H), 4.13 (d, 1H, *J* = 1.8 Hz), 3.69 (d, 2H, *J* = 1.8 Hz)
**4k**	δ (250 MHz, $CDCl_3$): 7.43-7.25 (m, 5H), 4.25 (d, 1H, *J* = 4.6 Hz), 4.00 (qd, 2H, *J* = 7.1 and 2.5 Hz), 3.80 (d, 1H, *J* = 4.6 Hz), 1.00 (t, 3H, *J* = 7.1 Hz)
**4l**	δ (250 MHz, $CDCl_3$): 7.41-7.19 (m, 5H), 4.81 (sept, 1H, *J* = 6.3 Hz), 4.23 (d, 1H, *J* = 4.6 Hz), 3.76 (d, 1H, *J* = 4.6 Hz), 1.30 (d, 3H, *J* = 6.3 Hz), 1.28 (d, 3H, *J* = 6.3 Hz)
**4m**	δ (250 MHz, $CDCl_3$): 7.38-7.27 (m, 5H), 5.84 (br s, 1H), 5.62 (br s, 1H), 4.32 (d, 1H, *J* = 4.8 Hz), 3.75 (d, 1H, *J* = 4.8 Hz)

Table S2. HPLC data for the epoxides.

Epoxide	Column	Eluent: *i*-PrOH/hexane	λ, nm	v, mL/min	T, °C	t_{R1}, min	t_{R2}, min
**4a**	Chiralcel OD-H	2:98	254	1.00	20	20.8	22.0
**4b**	Chiralcel OD-H	1:99	216	0.60	20	11.7	12.9
**4c**	Chiralcel OD-H	1:99	216	0.40	20	13.3	18.6
**4d**	Chiralcel OB-H	10:90	216	1.00	20	12.0	16.2
**4e**	Chiralcel OB-H	2:98	216	0.70	25	14.8	18.7
**4f**	Chiralcel OJ-H	30:70	254	1.00	20	12.6	22.3
**4g**	Chiralpak AD-H	8:92	254	0.80	20	21.5	22.6
**4h**	Chiralcel OD-H	10:90	216	0.80	20	12.9	18.4
**4i**	Chiralpak AD-H	5:95	216	1.00	40	5.9	6.7
**4j**	Chiralpak AS-3	25:75	210	0.72	35	52.0	55.3
**4k**	Chiralpak AD-H	2:98	216	1.00	25	7.4	8.2
**4l**	Chiralcel OJ-H	10:90	216	1.00	25	10.6	11.2
**4m**	Chiralcel OJ-H	10:90	210	0.88	20	22.7	27.2

Figure S1. Selected examples of ^{1}H NMR spectra of reaction mixtures.

The most common admixtures were diethyl ether (q 3.47 ppm, t 1.21 ppm), CH_3CN (s 1.96 ppm) and EBA (broad signals from 1.8 to 0.8 ppm)

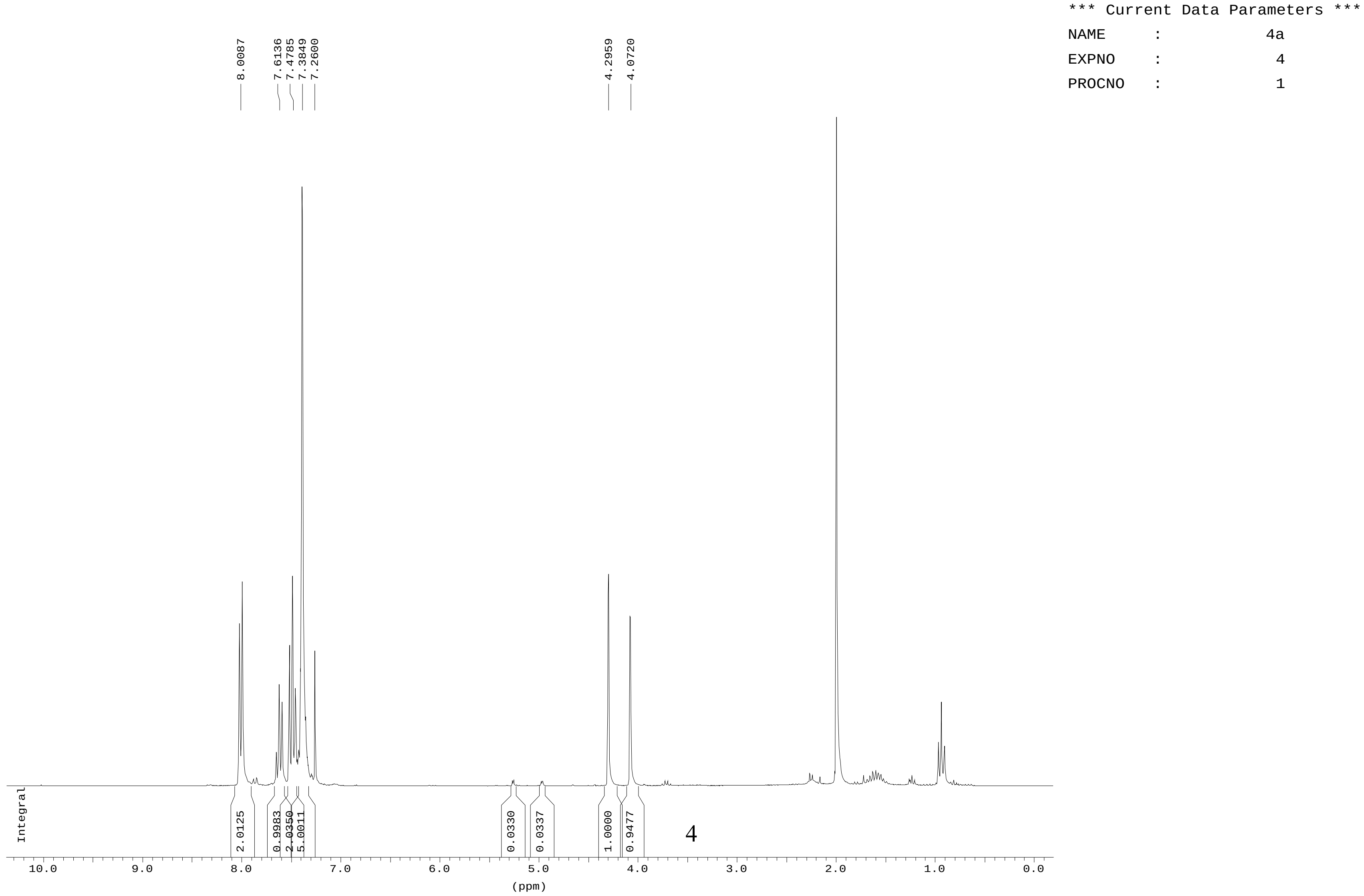

*** Current Data Parameters ***

NAME : 4b

EXPNO : 1

PROCNO : 1

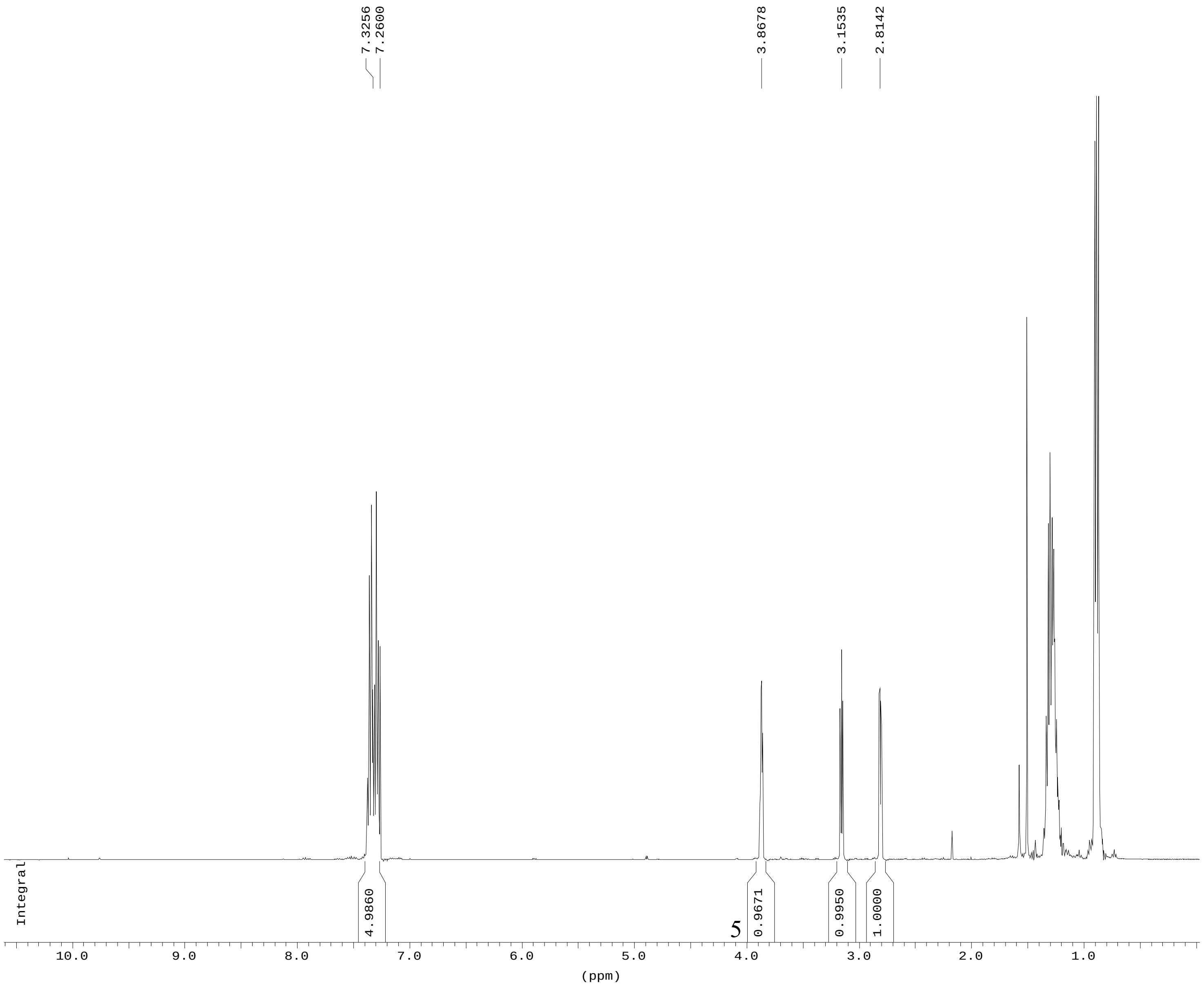

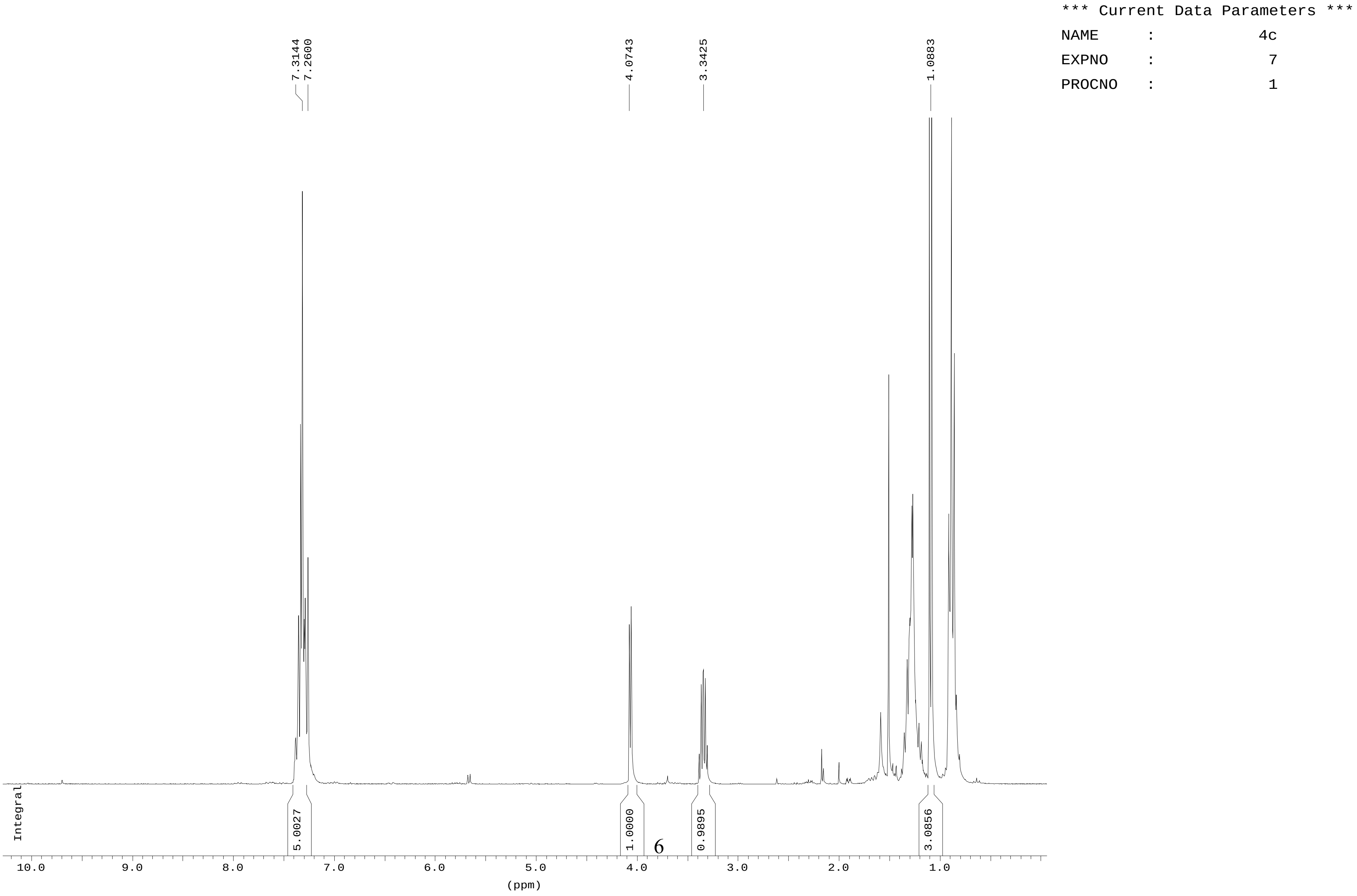
*** Current Data Parameters ***
NAME : 4c
EXPNO : 7
PROCNO : 1
7.3144
7.2600
4.0743
3.3425
1.0883
Integral
5.0027
1.0000
0.9895
3.0856
10.0 9.0 8.0 7.0 6.0 5.0 4.0 3.0 2.0 1.0
(ppm)

*** Current Data Parameters ***

NAME : 4d

EXPNO : 13

PROCNO : 1

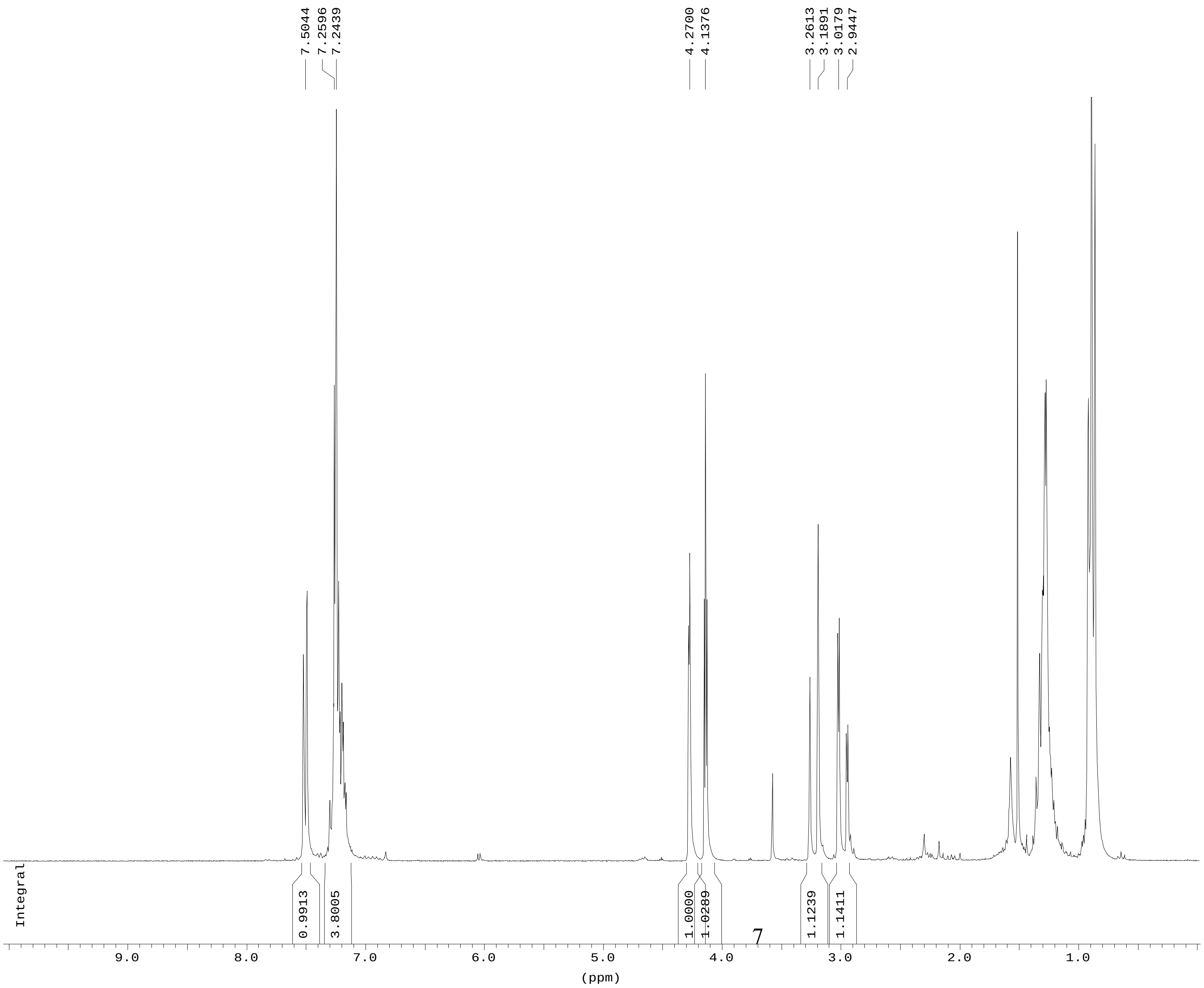

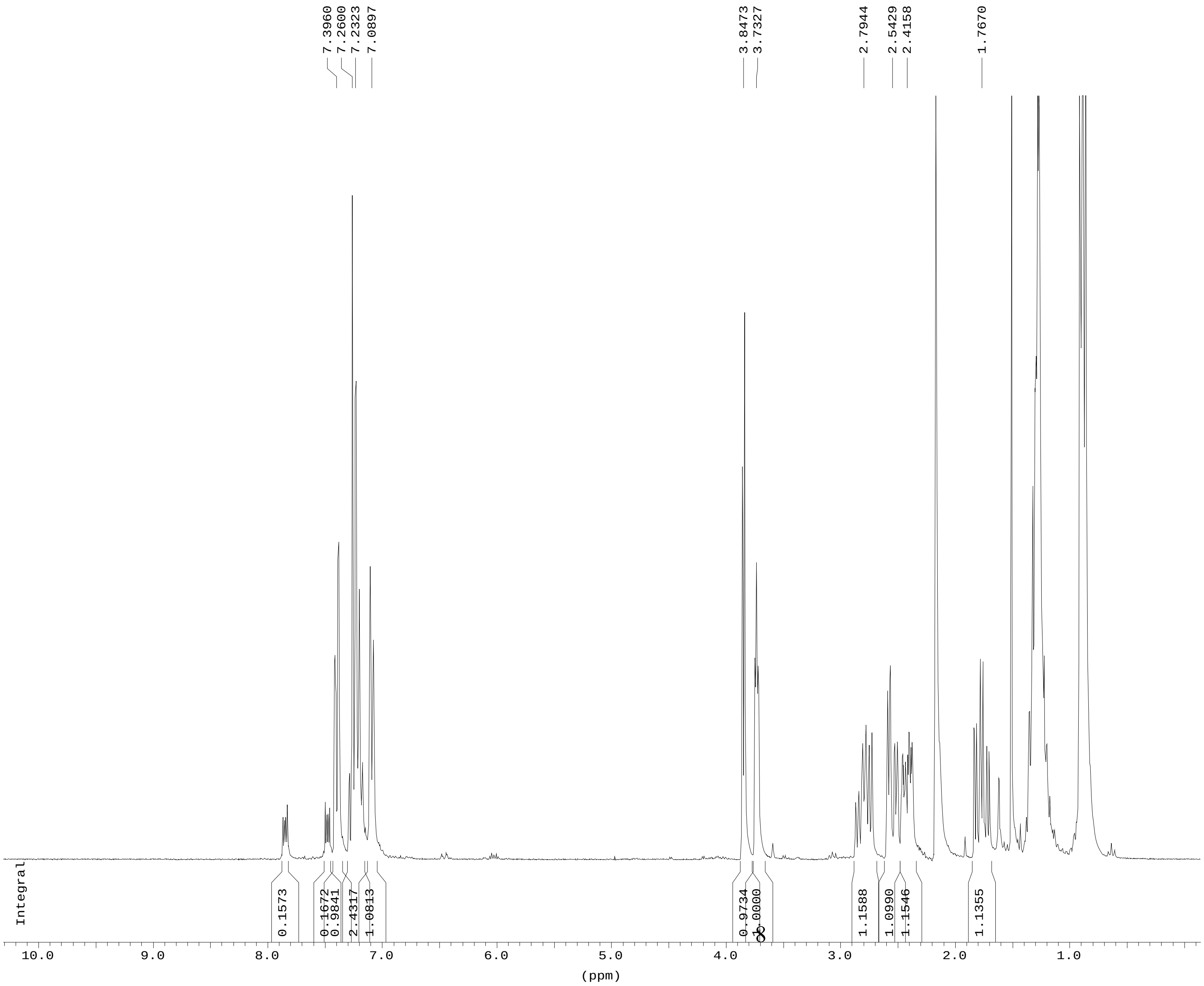

*** Current Data Parameters ***

NAME : 4e

EXPNO : 8

PROCNO : 1

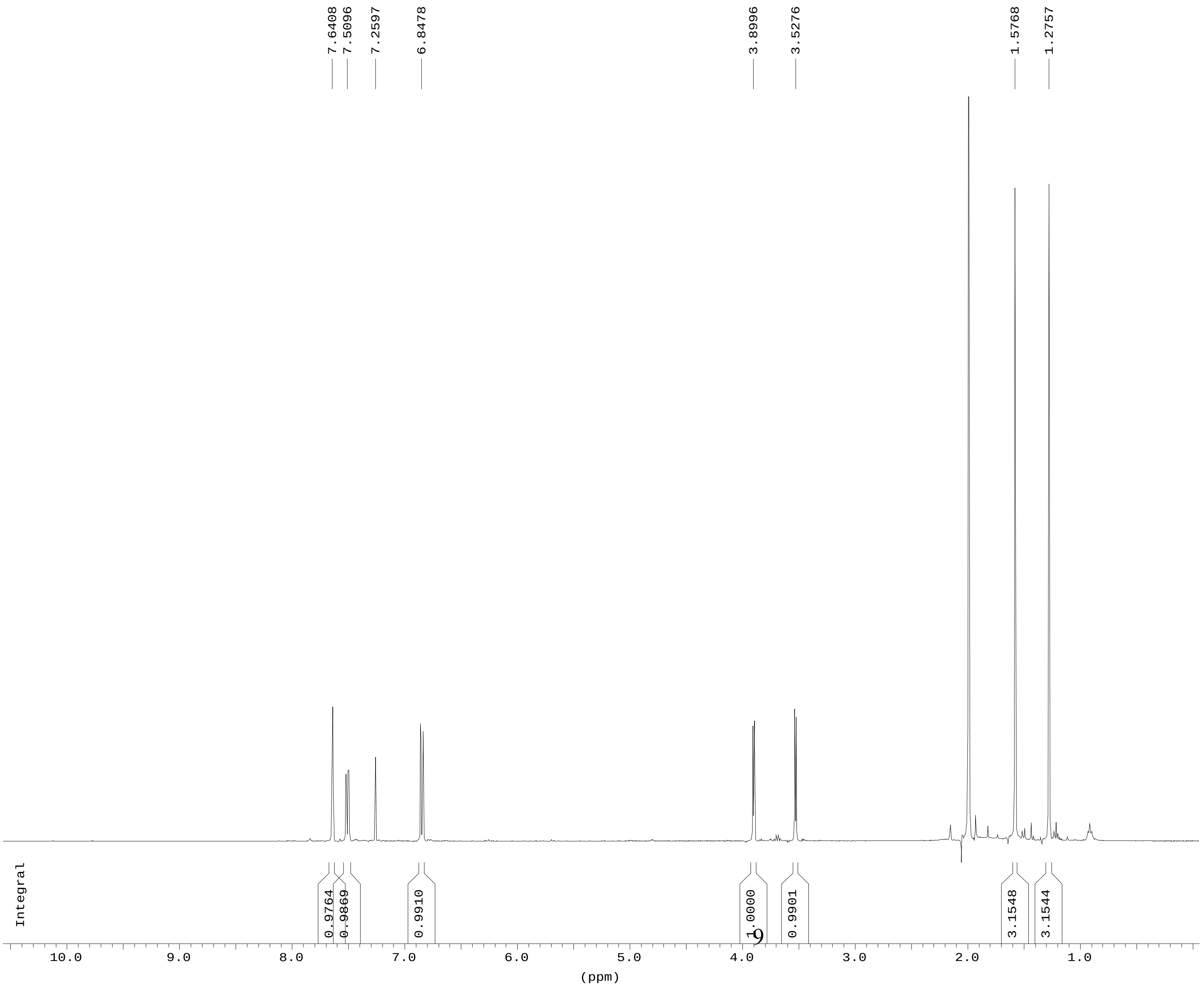

*** Current Data Parameters ***

NAME : 4f

EXPNO : 2

PROCNO : 1

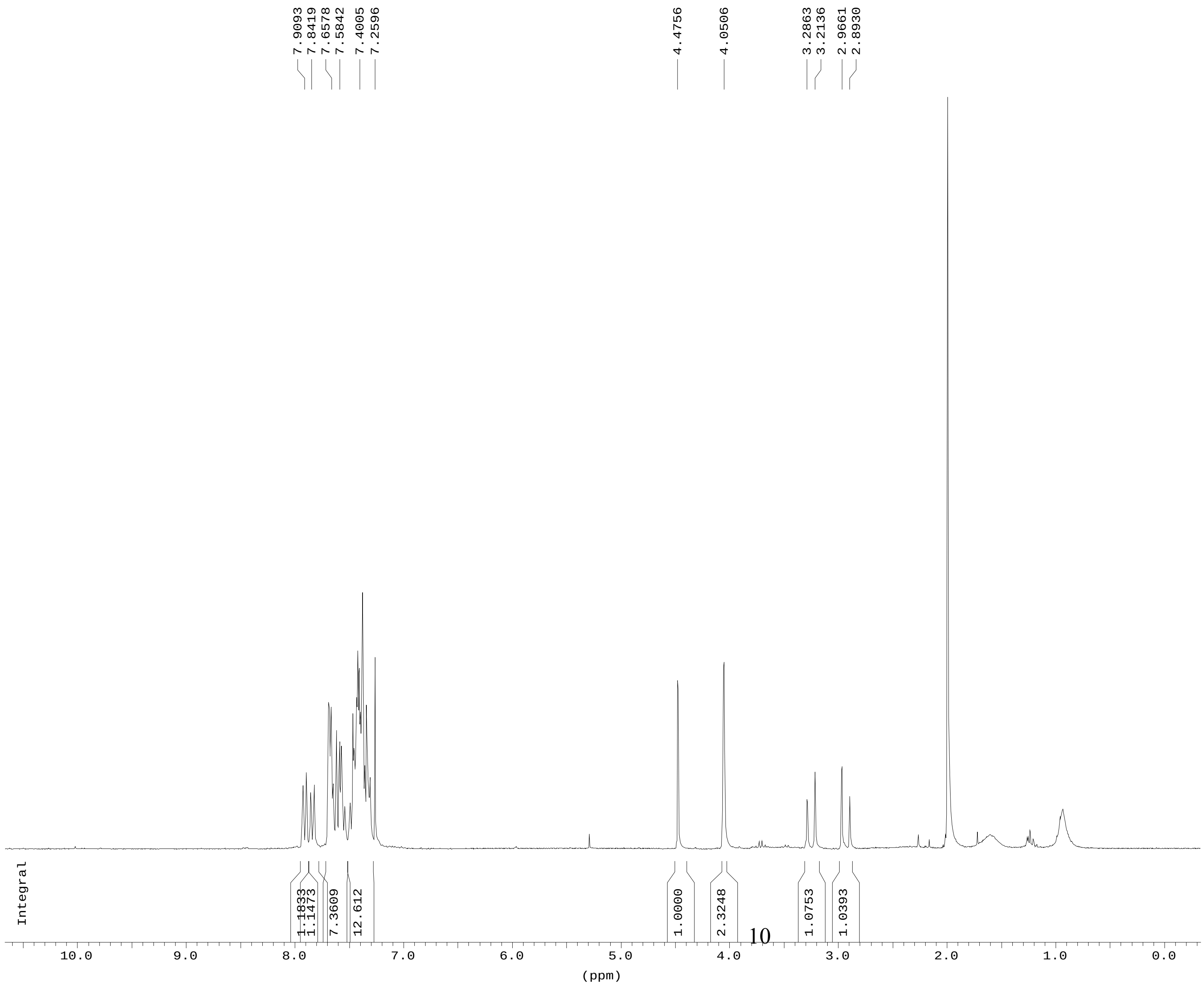

*** Current Data Parameters ***

NAME : 4g

EXPNO : 14

PROCNO : 1

*** Current Data Parameters ***

NAME : 4h

EXPNO : 10

PROCNO : 1

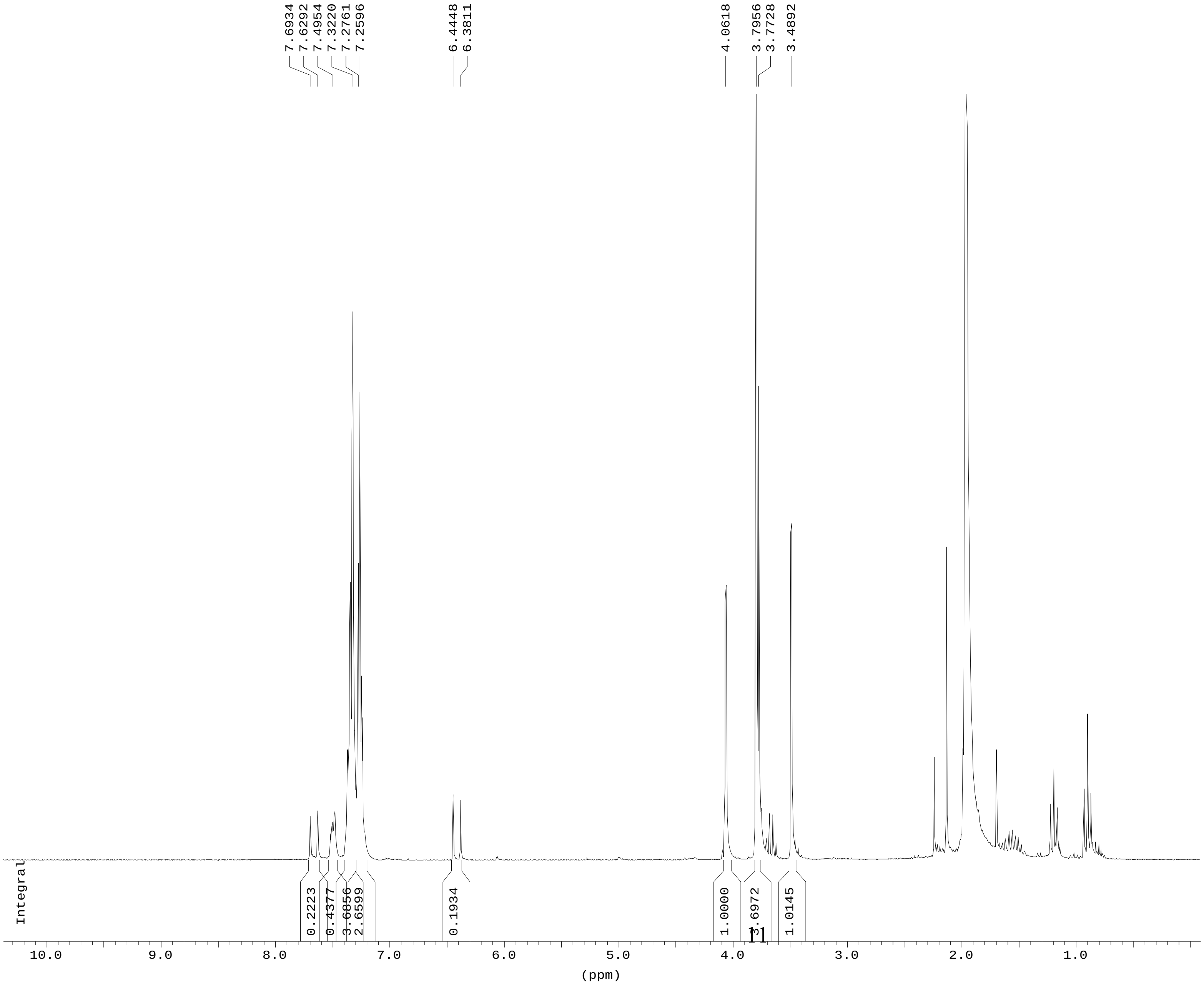

*** Current Data Parameters ***

NAME : 4i

EXPNO : 9

PROCNO : 1

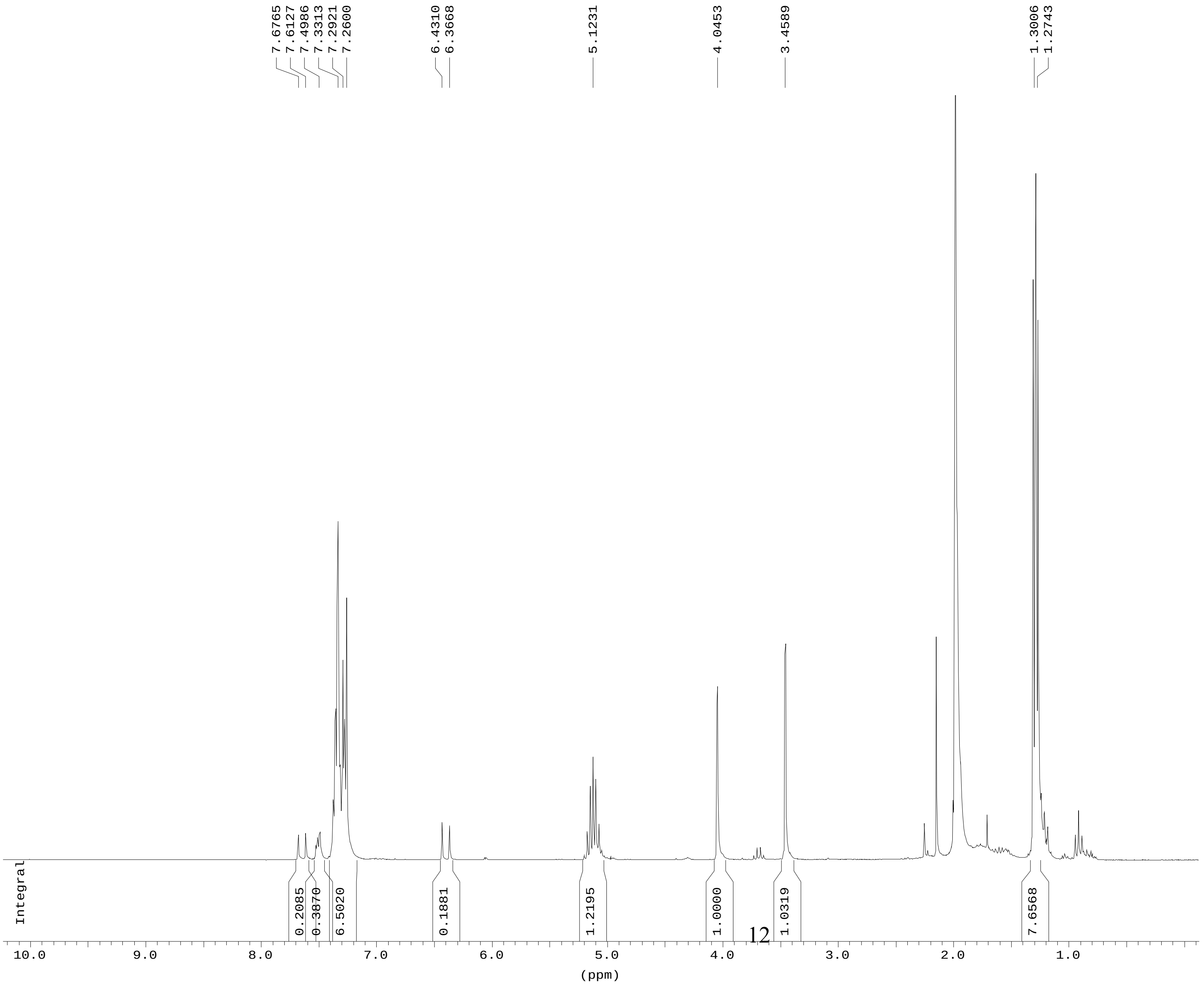

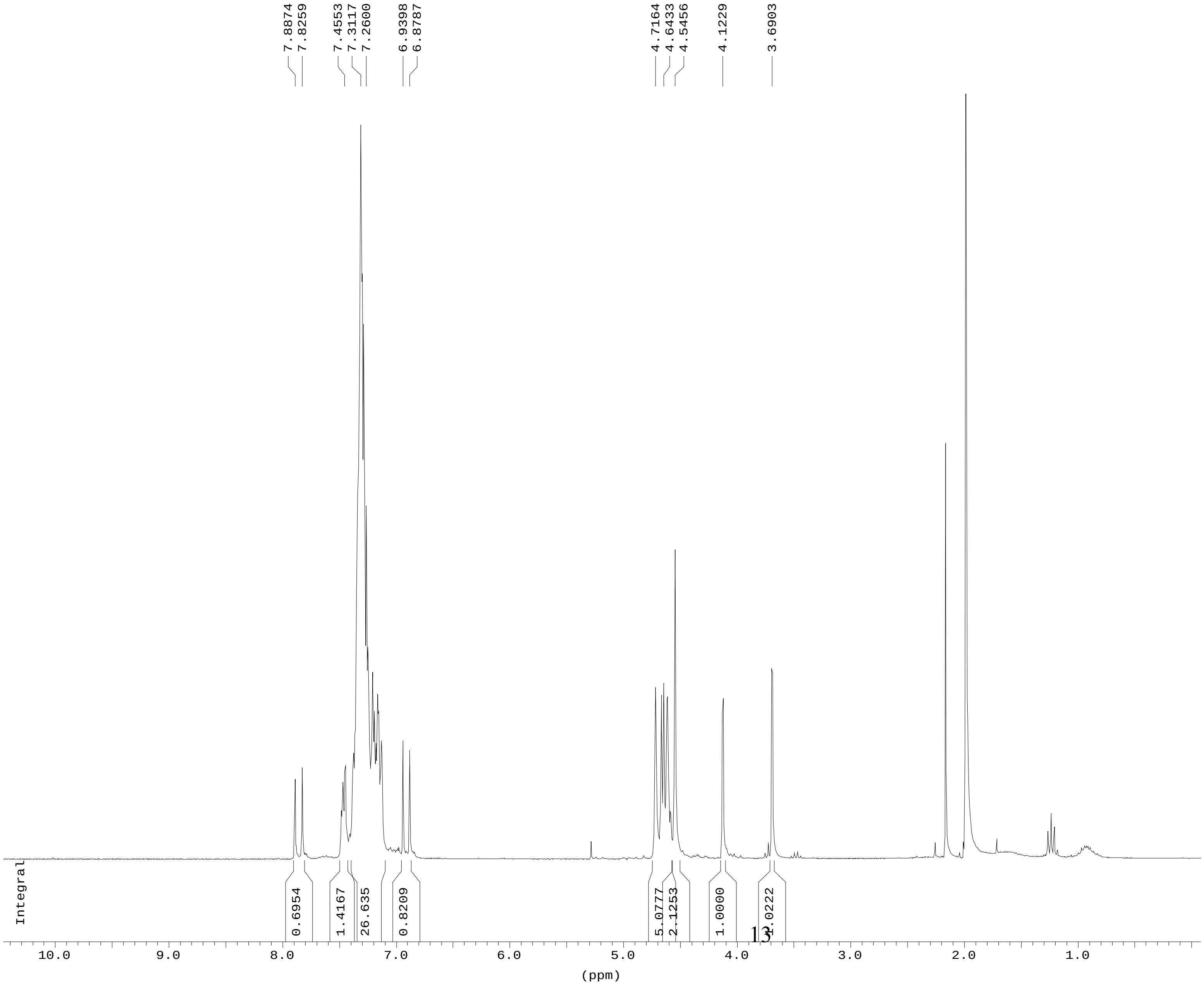

*** Current Data Parameters ***

NAME : 4j

EXPNO : 16

PROCNO : 1

*** Current Data Parameters ***
NAME : 4k
EXPNO : 6
PROCNO : 1

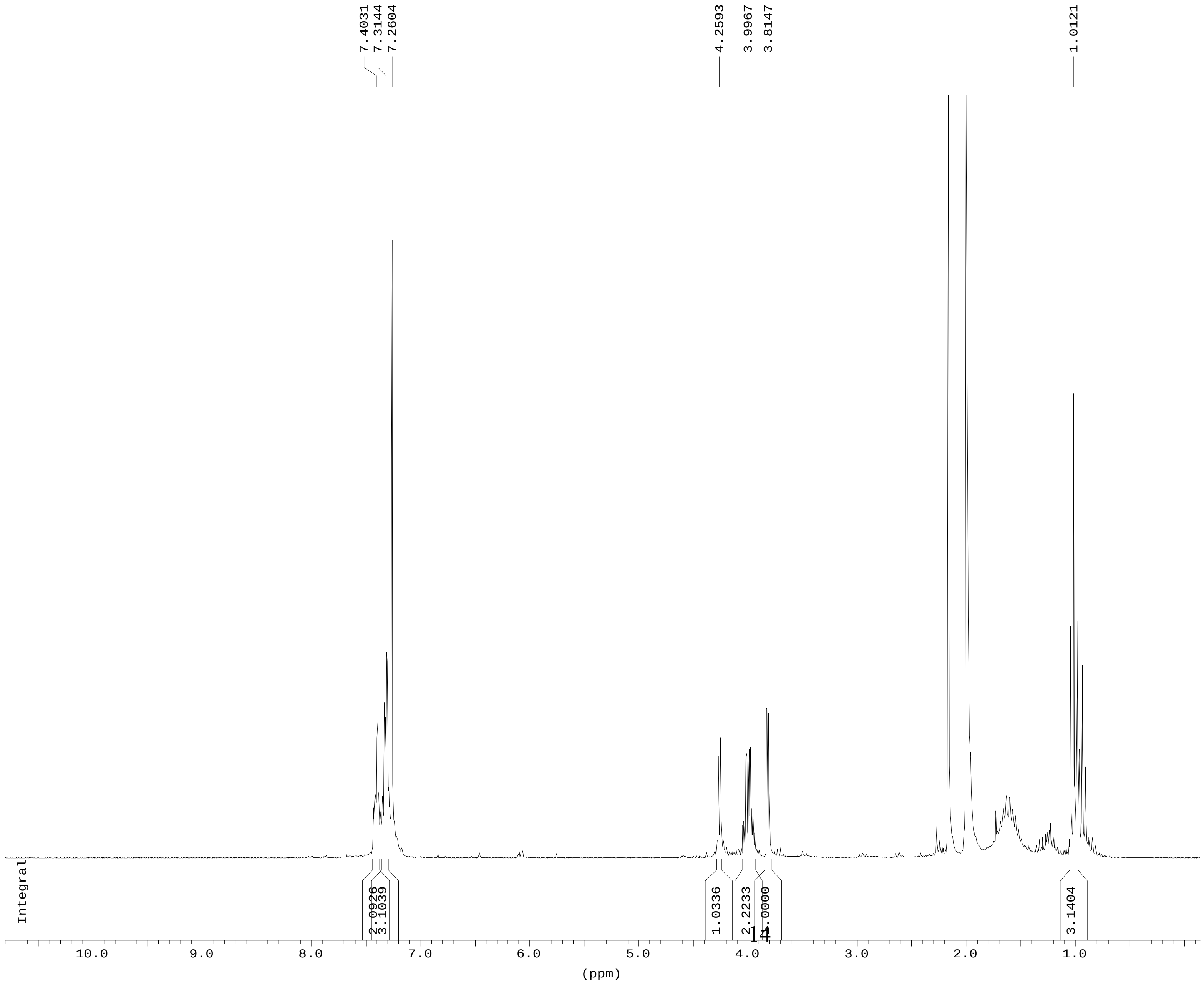

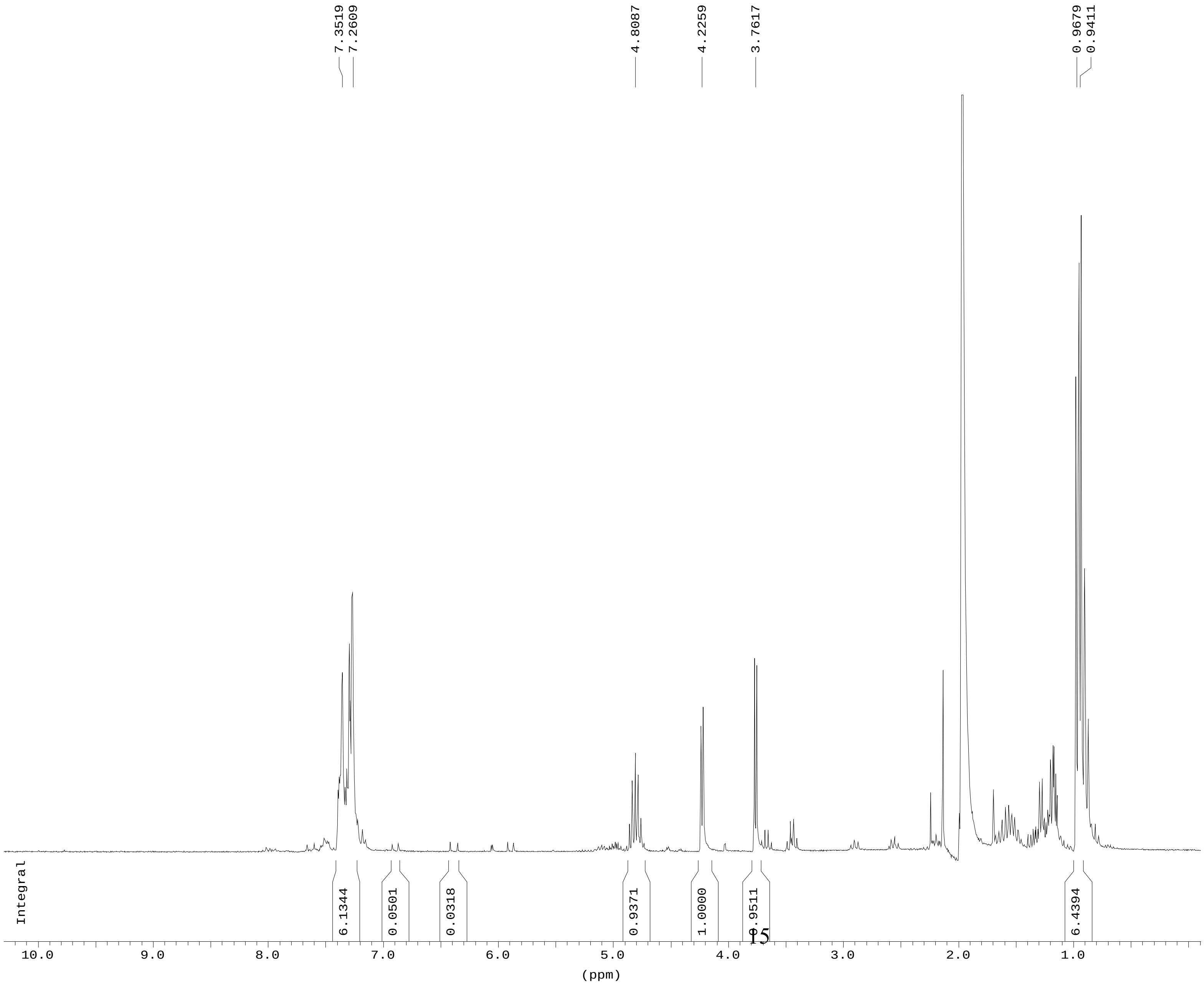

*** Current Data Parameters ***

NAME : 4l

EXPNO : 5

PROCNO : 1

*** Current Data Parameters ***

NAME : 4m

EXPNO : 15

PROCNO : 1

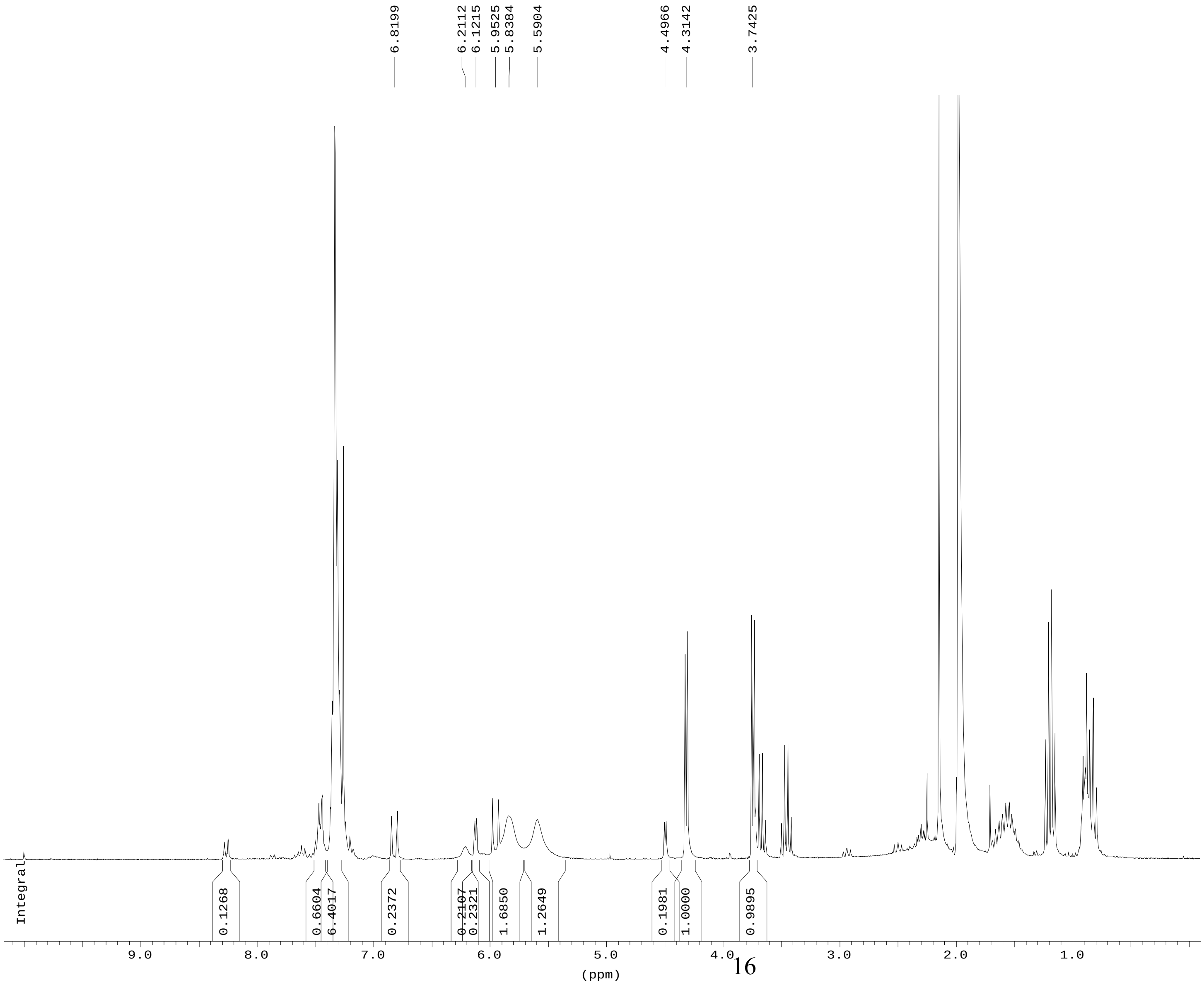

Figure S2. Selected examples of chiral HPLC chromatograms of reaction mixtures.

4a

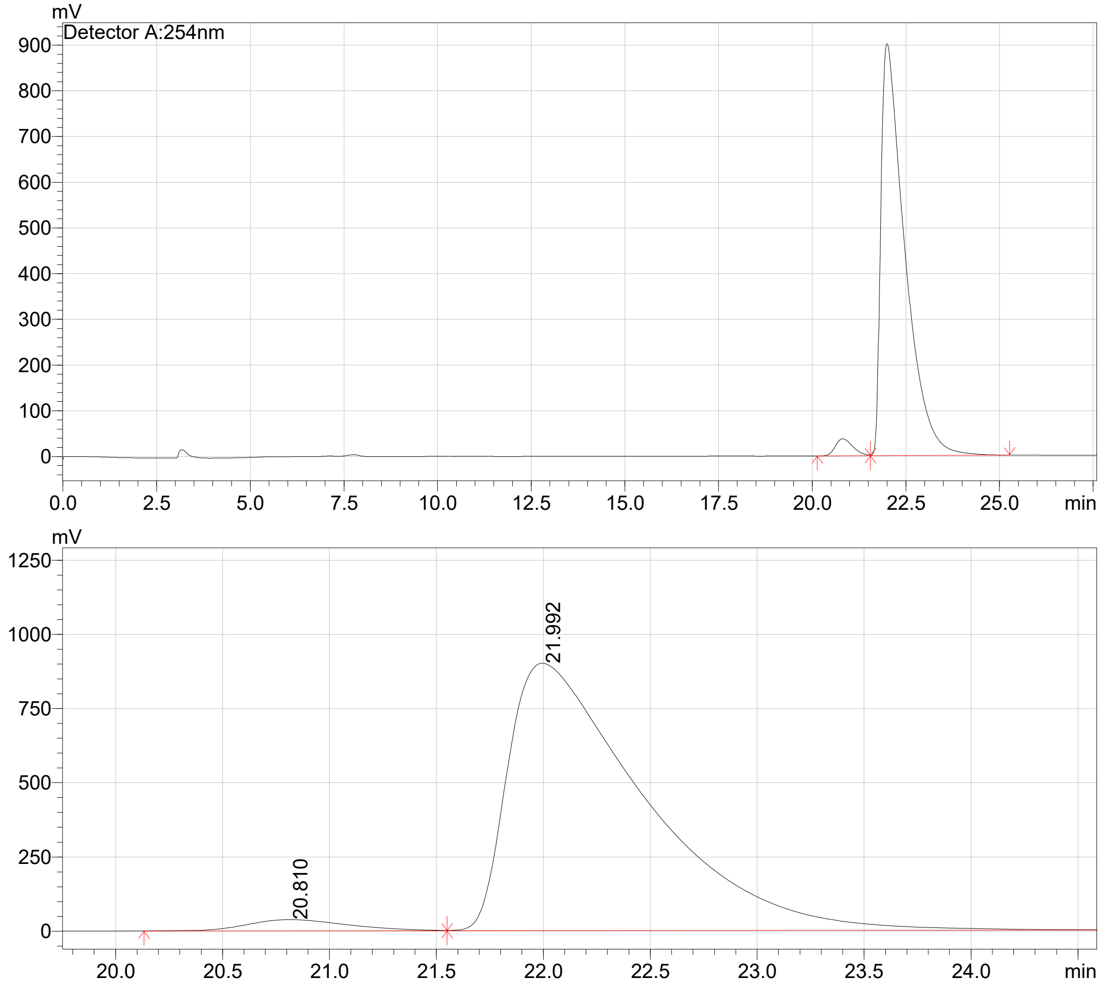

4a (racemate)

Peak Table

Peak#	Ret. Time	Area	Area%	Height	Peak Start	Peak End
1	19.105	8008540	49.8602	234807	18.550	20.350
2	20.870	8053460	50.1398	212809	20.350	23.533

Detector A

Close Help

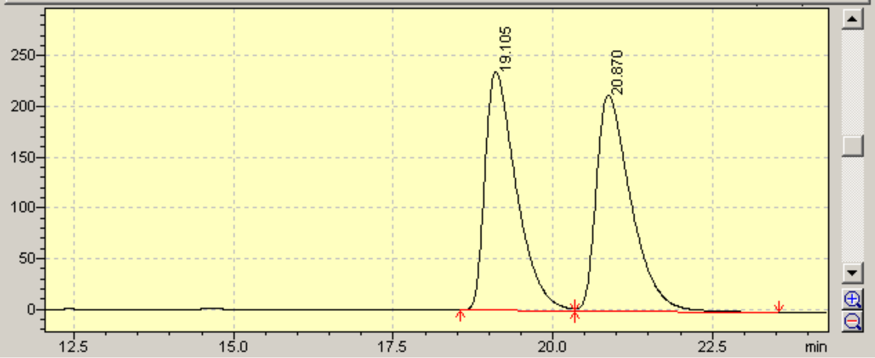

4b

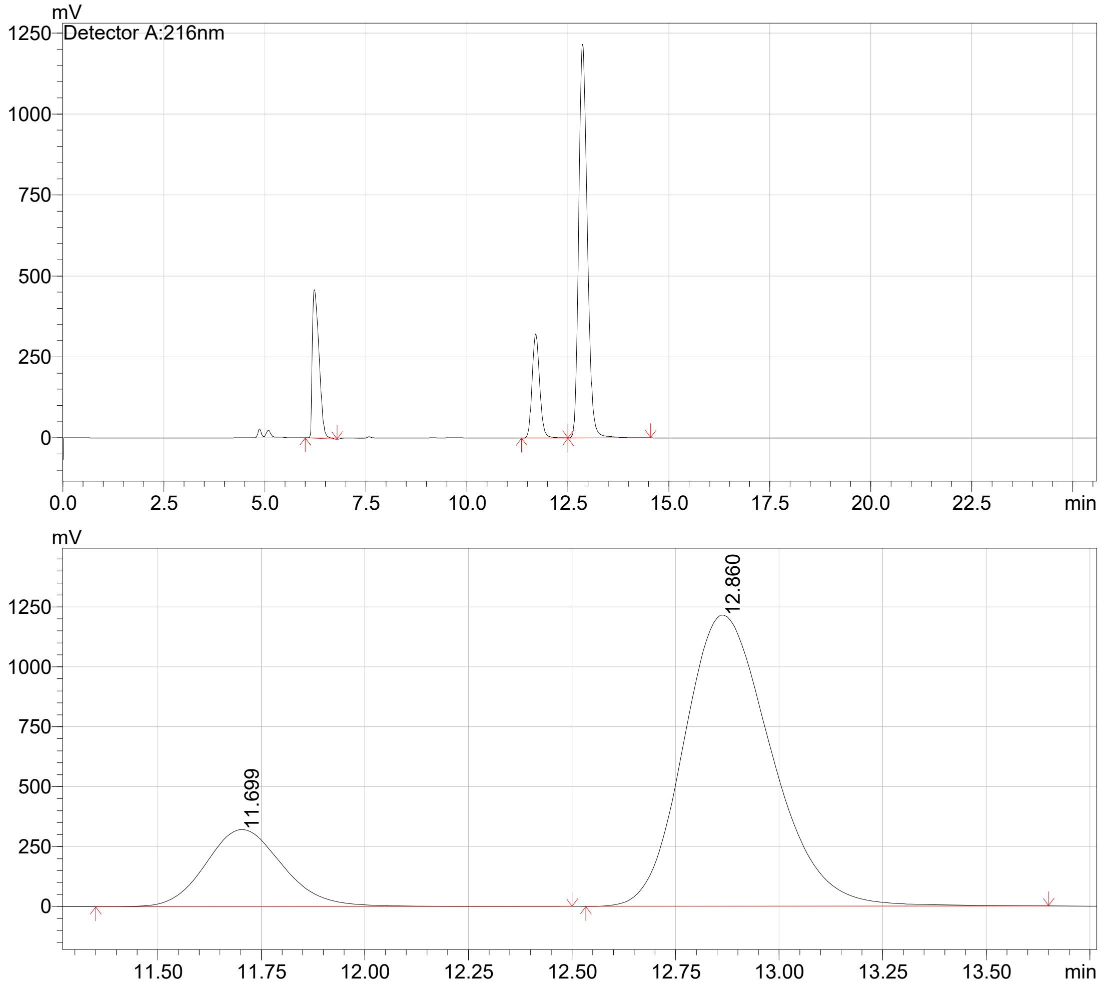

4b (racemate)

Peak Table

Peak#	Ret. Time	Area	Area%	Height	Peak Start	Peak End
1	11.640	28294430	50.2697	1324166	11.100	12.283
2	12.749	27990790	49.7303	1325266	12.283	13.467

Detector A

Close Help

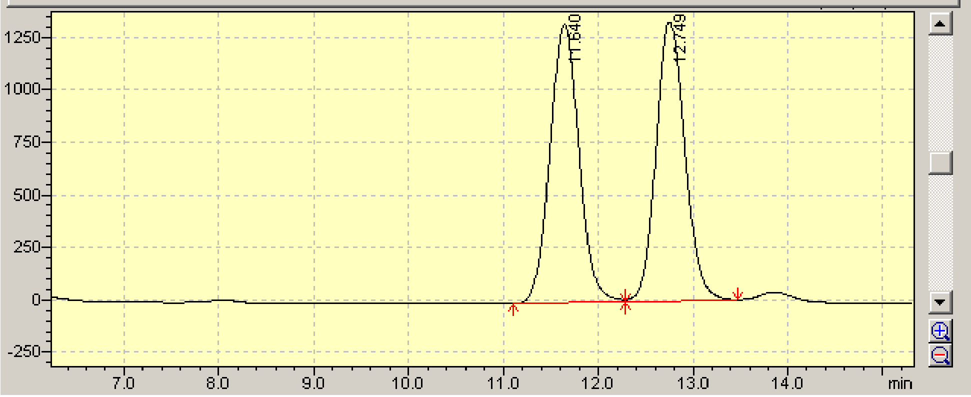

4c

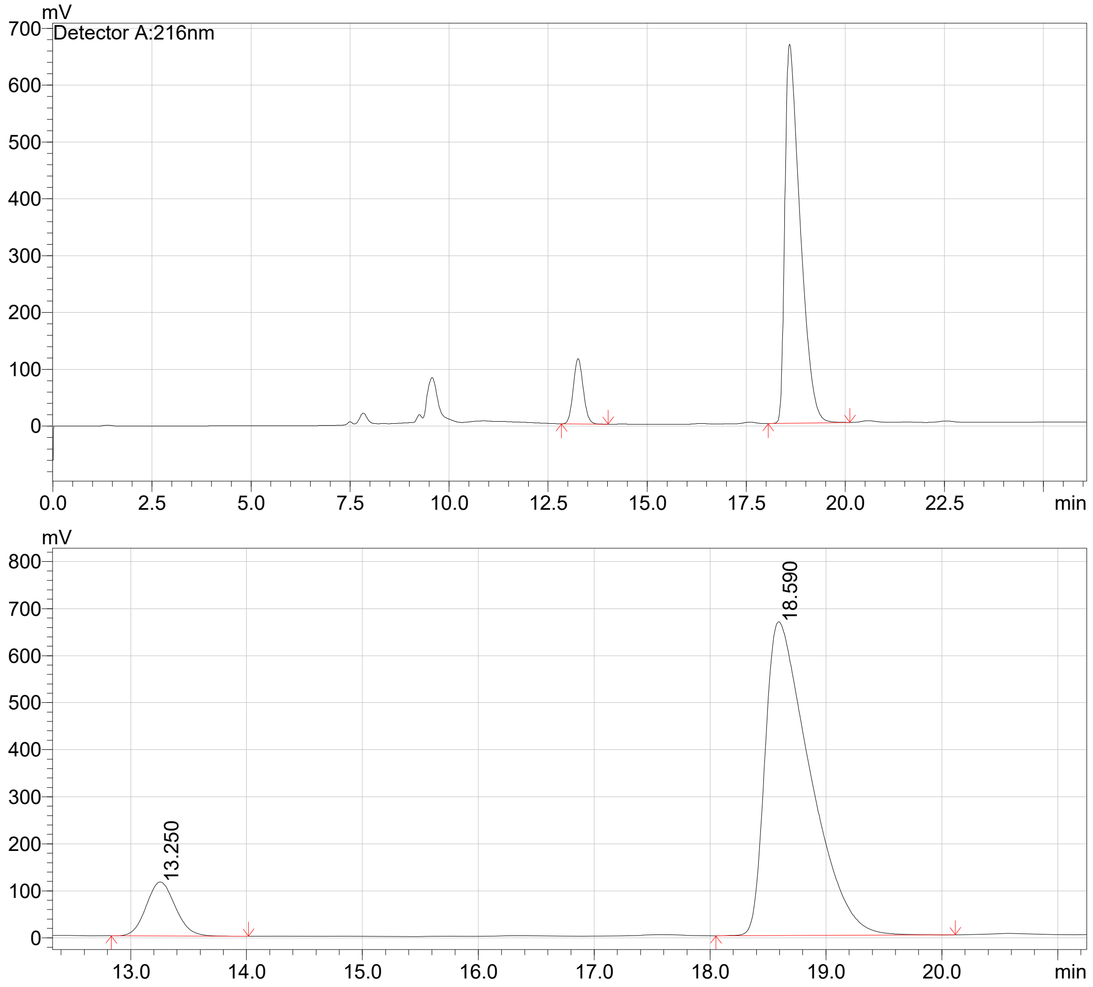

4c (racemate)

Peak Table

Peak#	Ret. Time	Area	Area%	Height	Peak Start	Peak End
1	13.125	3722655	49.8796	101060	12.283	14.483
2	18.416	3740622	50.1204	121902	17.833	19.050

Detector A

Close Help

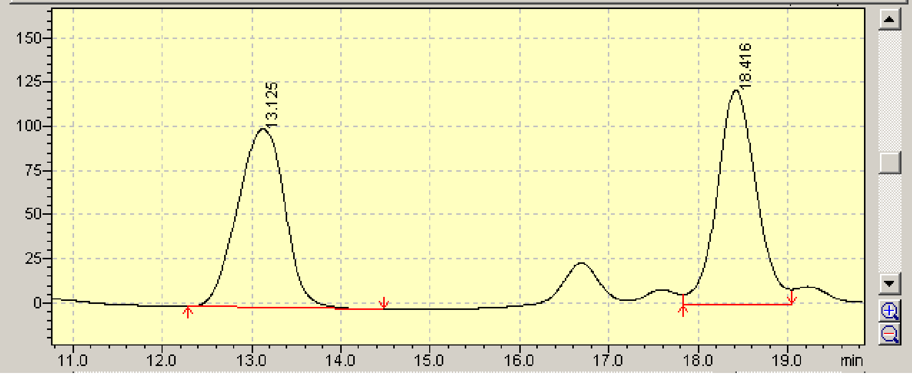

4d

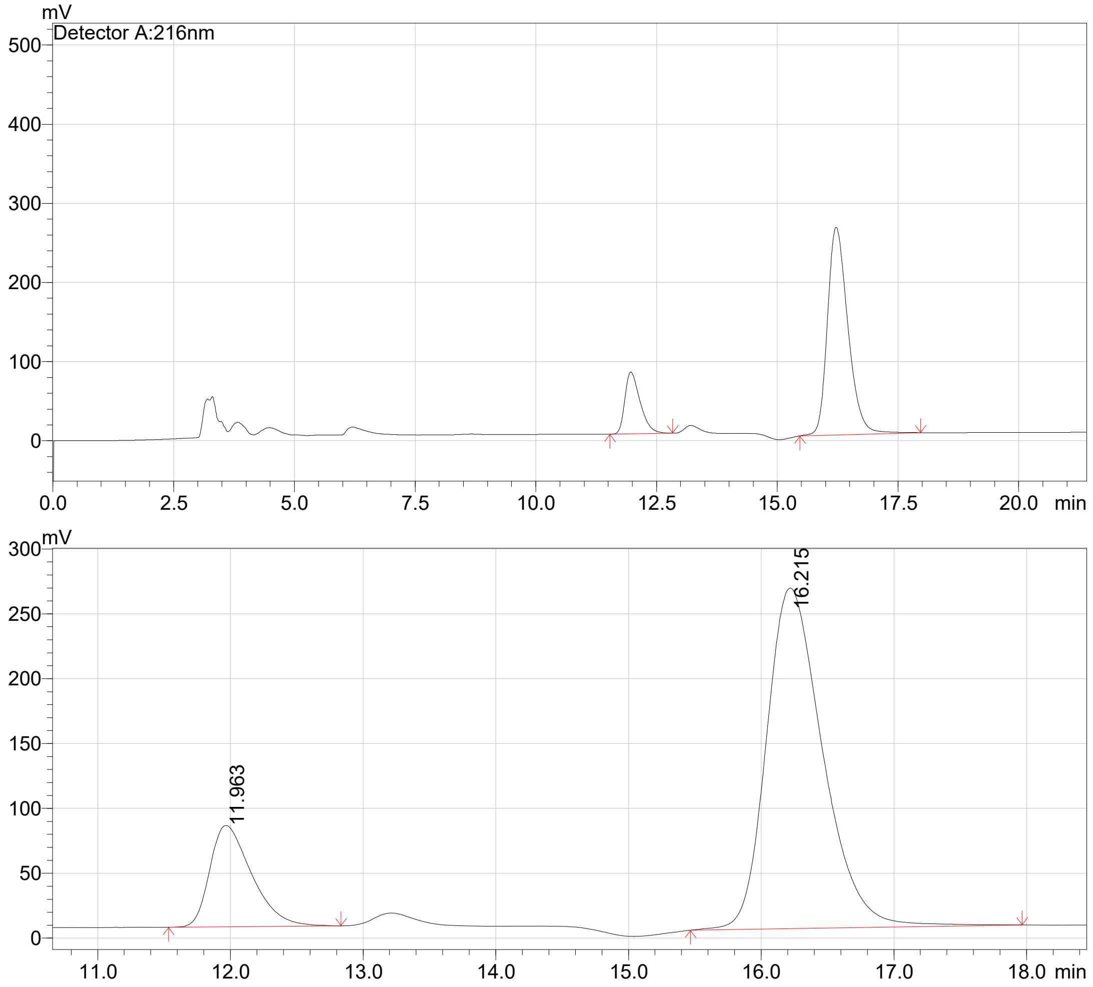

4d (racemate)

Peak Table

Peak#	Ret. Time	Area	Area%	Height	Peak Start	Peak End
1	13.253	772315	53.4632	31865	12.867	13.650
2	18.426	672259	46.5368	15756	17.700	19.933

Detector A

Close Help

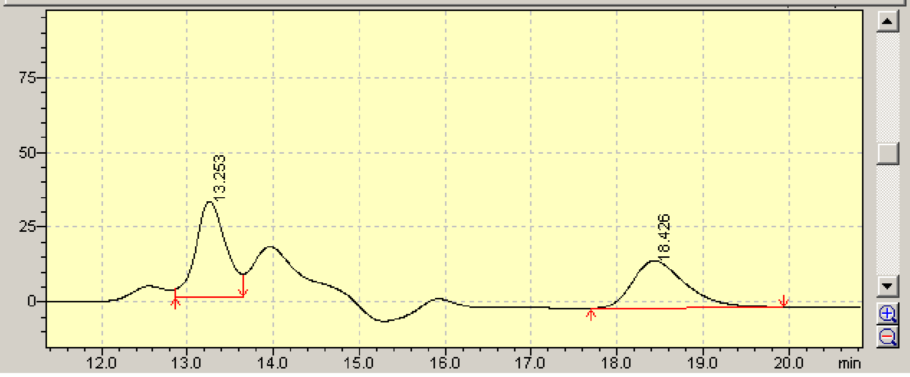

4e

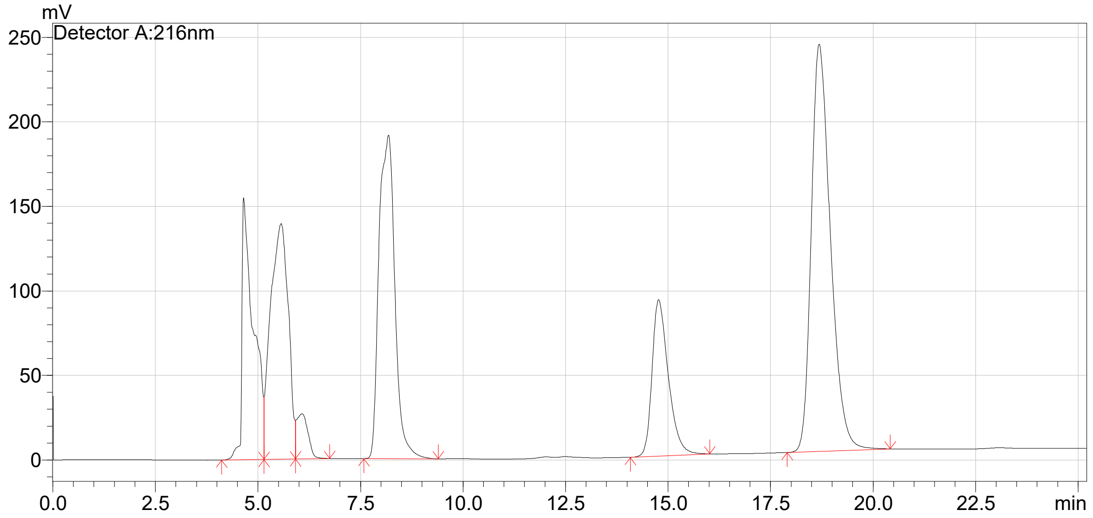
mV
Detector A:216nm
250
200
150
100
50
0
0.0
2.5
5.0
7.5
10.0
12.5
15.0
17.5
20.0
22.5
min

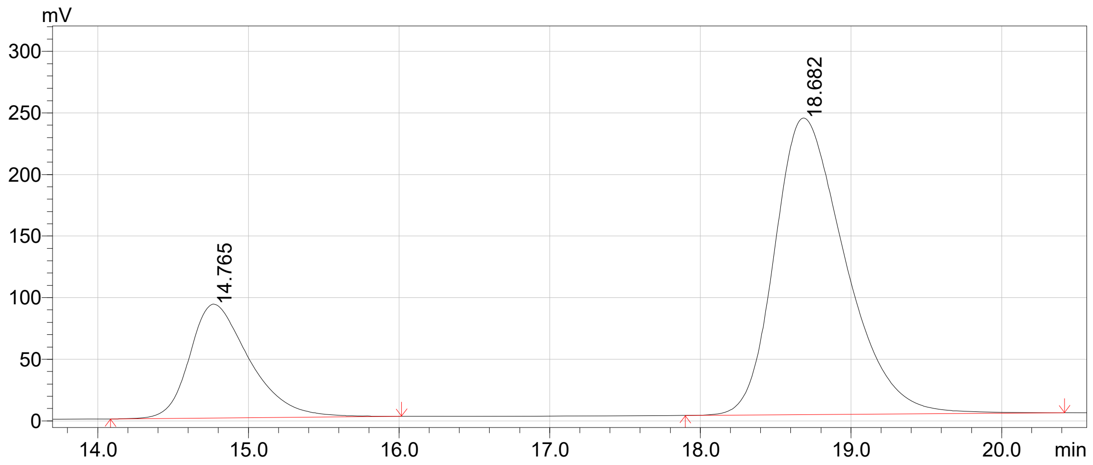
mV
300
250
200
150
100
50
0
14.765
18.682
14.0
15.0
16.0
17.0
18.0
19.0
20.0
min

4f

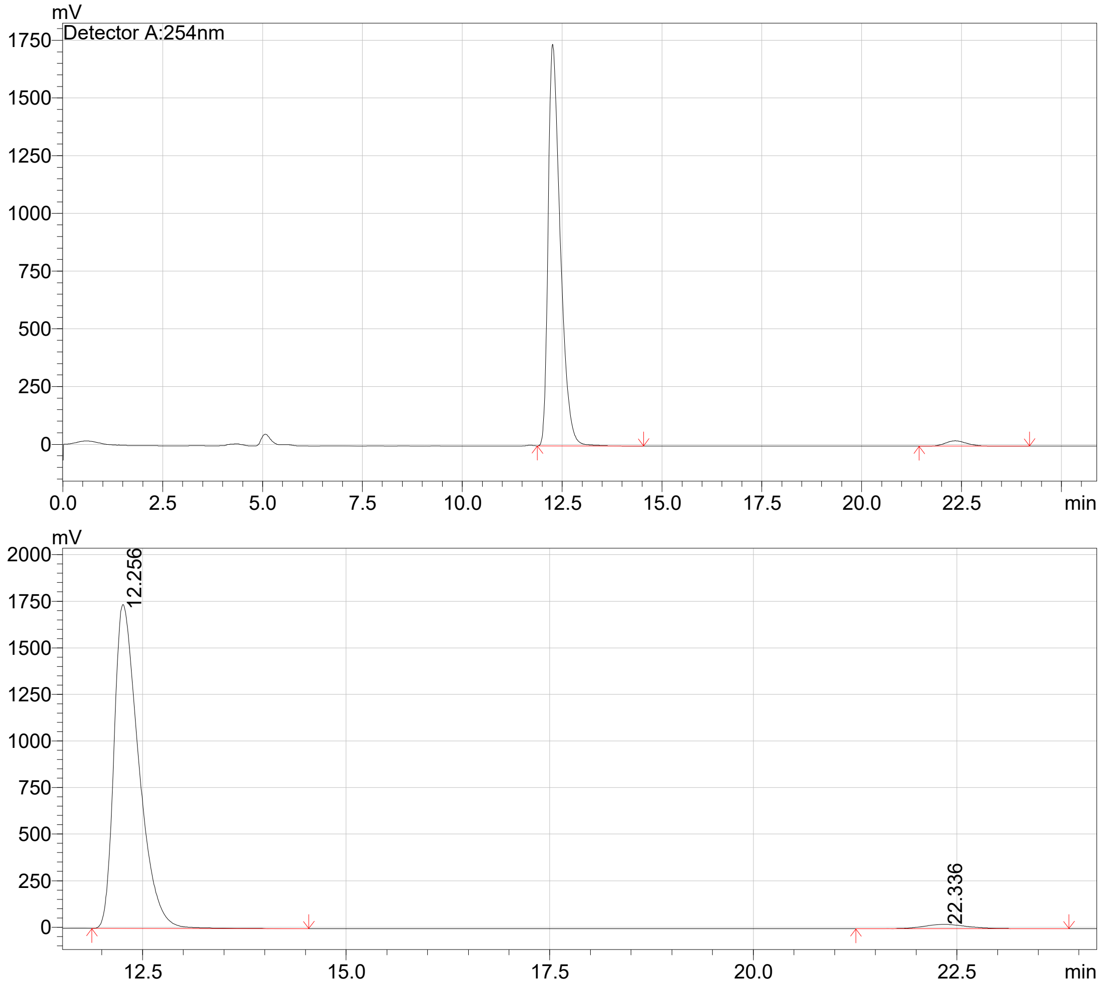

4f (racemate)

Peak Table

Peak#	Ret. Time	Area	Area%	Height	Peak Start	Peak End
1	12.188	4738461	49.6545	254364	11.825	12.725
2	22.003	4804399	50.3455	128740	21.042	24.358

Detector A

Close Help

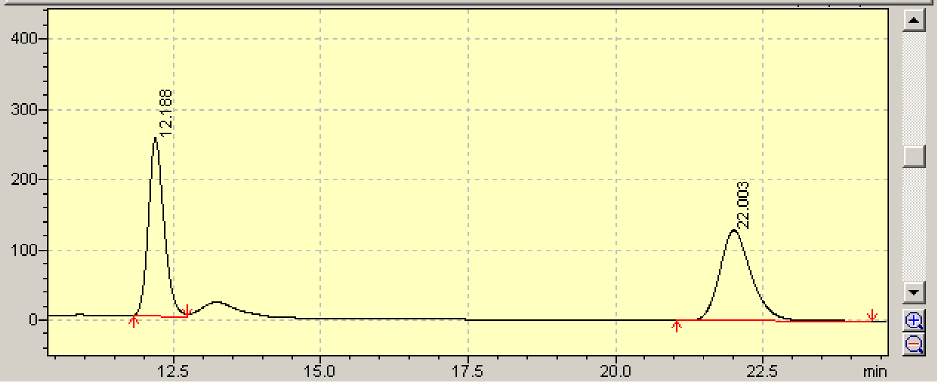

4g

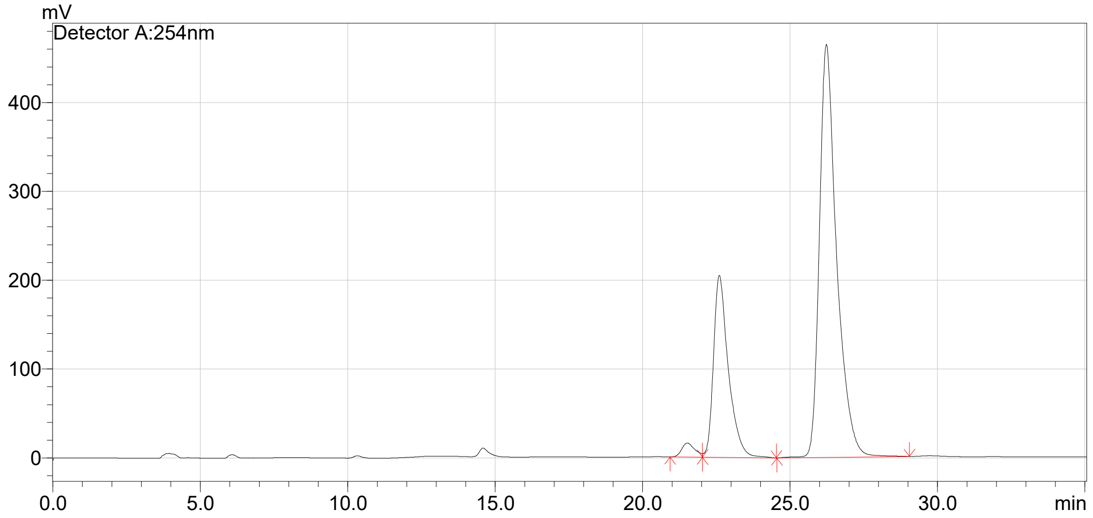
mV
Detector A:254nm
400
300
200
100
0
0.0
5.0
10.0
15.0
20.0
25.0
30.0
min

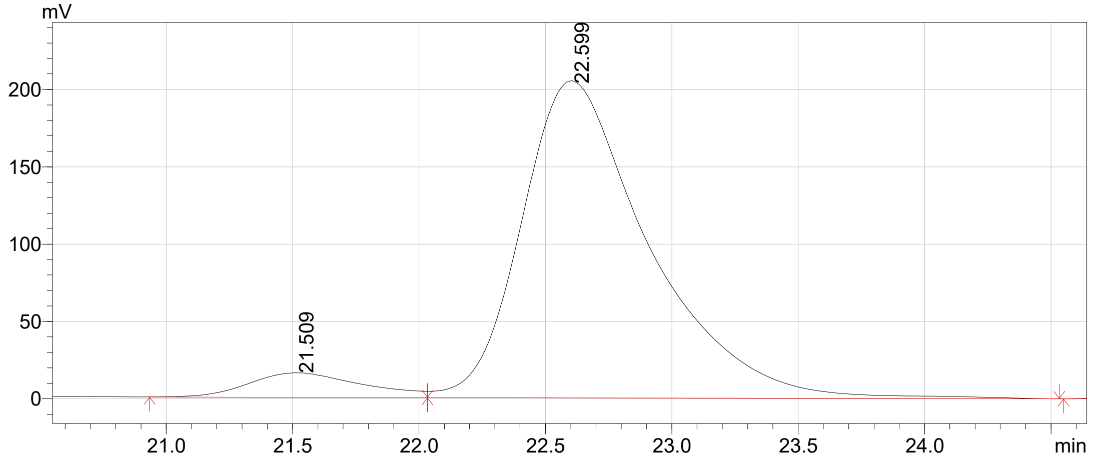
mV
22.599
21.509
200
150
100
50
0
21.0
21.5
22.0
22.5
23.0
23.5
24.0
min

4h

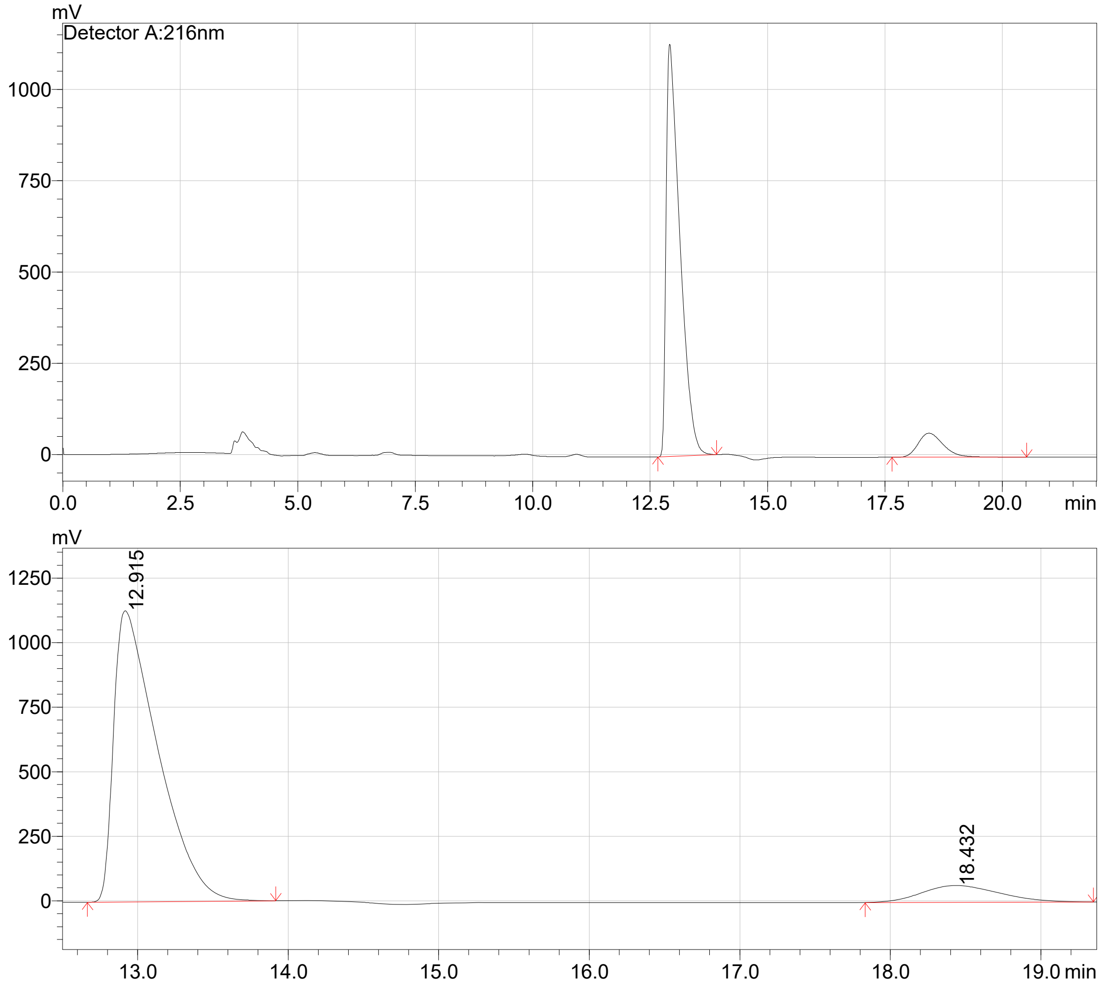

4h (racemate)

Peak Table

Peak#	Ret. Time	Area	Area%	Height	Peak Start	Peak End
1	13.482	14431512	50.0948	667370	13.217	14.283
2	18.977	14376881	49.9052	507824	17.433	20.317

Detector A

Close Help

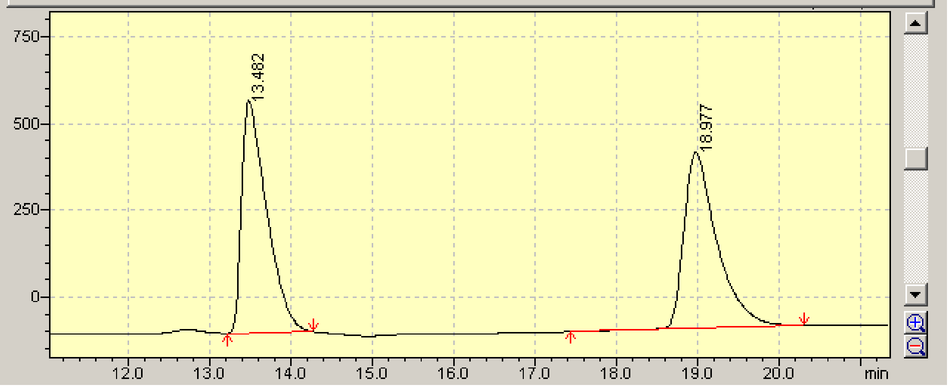

4i

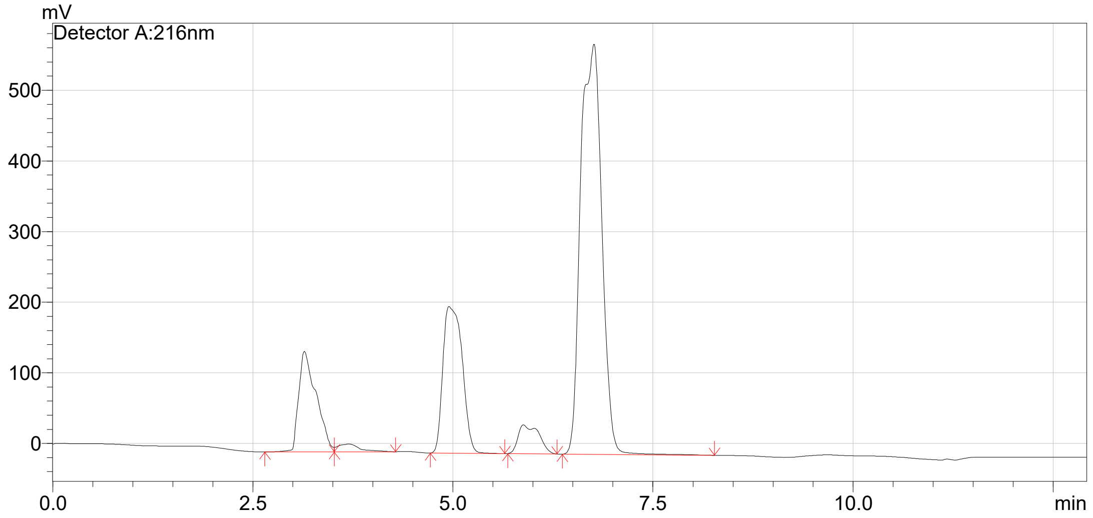
mV
Detector A:216nm
500
400
300
200
100
0
0.0
2.5
5.0
7.5
10.0
min

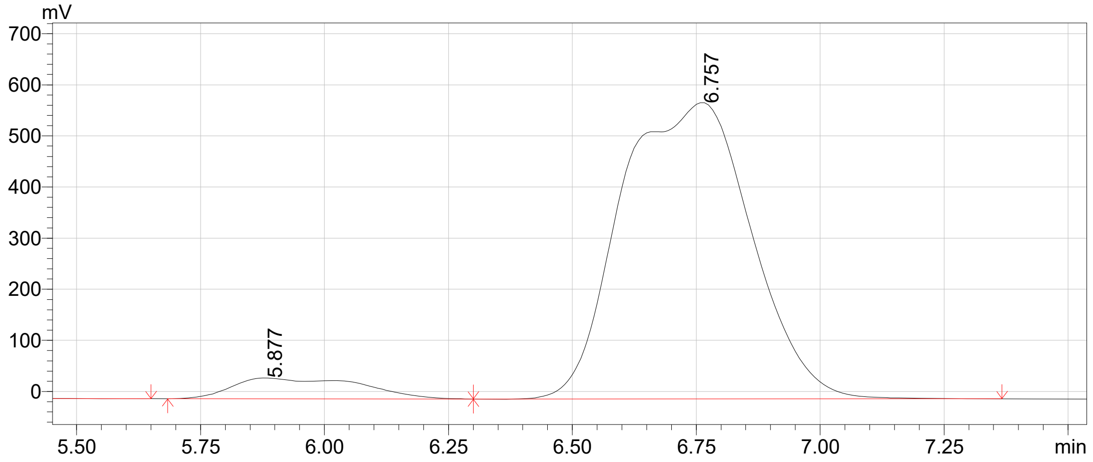
mV
700
600
500
400
300
200
100
0
5.877
6.757
5.50
5.75
6.00
6.25
6.50
6.75
7.00
7.25
min

4j

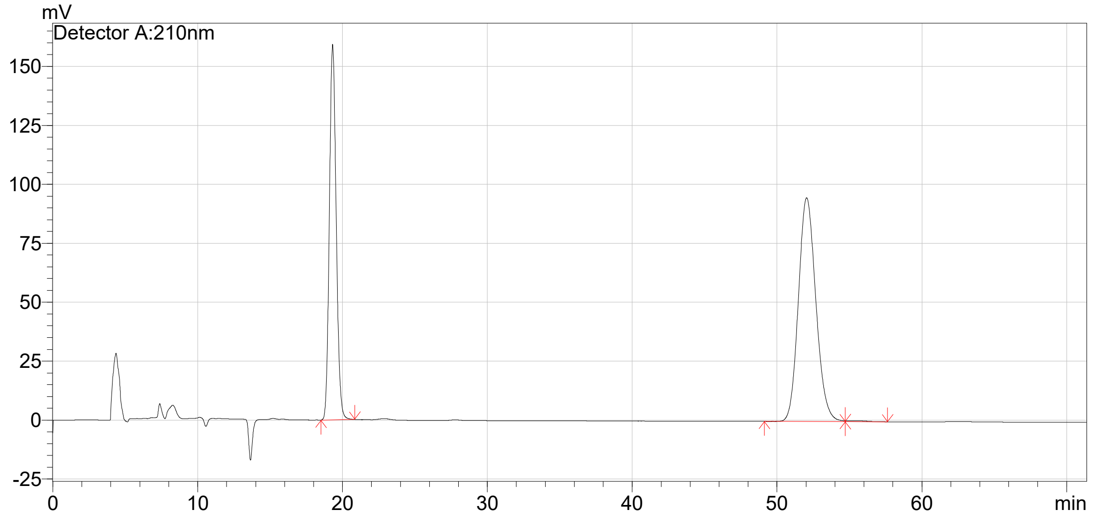
mV
Detector A:210nm
150
125
100
75
50
25
0
-25
0
10
20
30
40
50
60
min

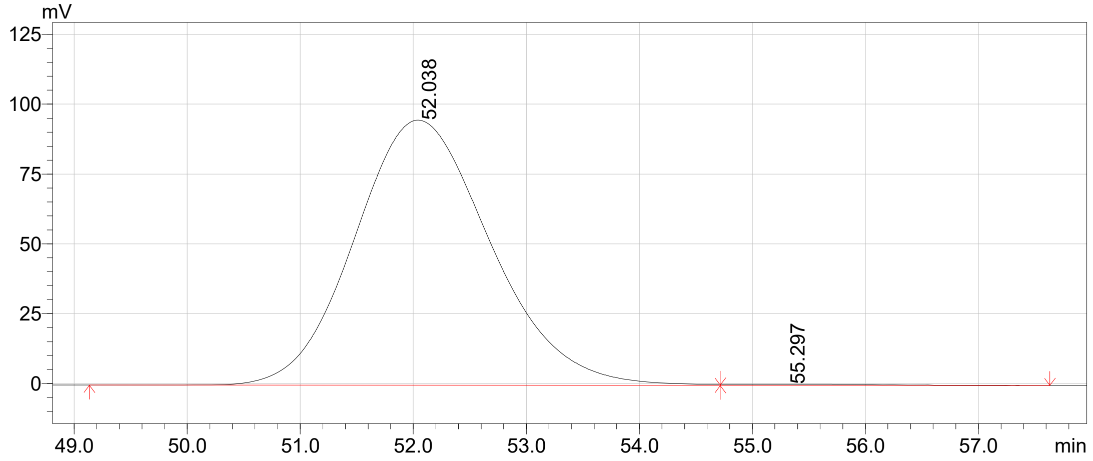
mV
125
100
75
50
25
0
52.038
55.297
49.0
50.0
51.0
52.0
53.0
54.0
55.0
56.0
57.0
min

4k

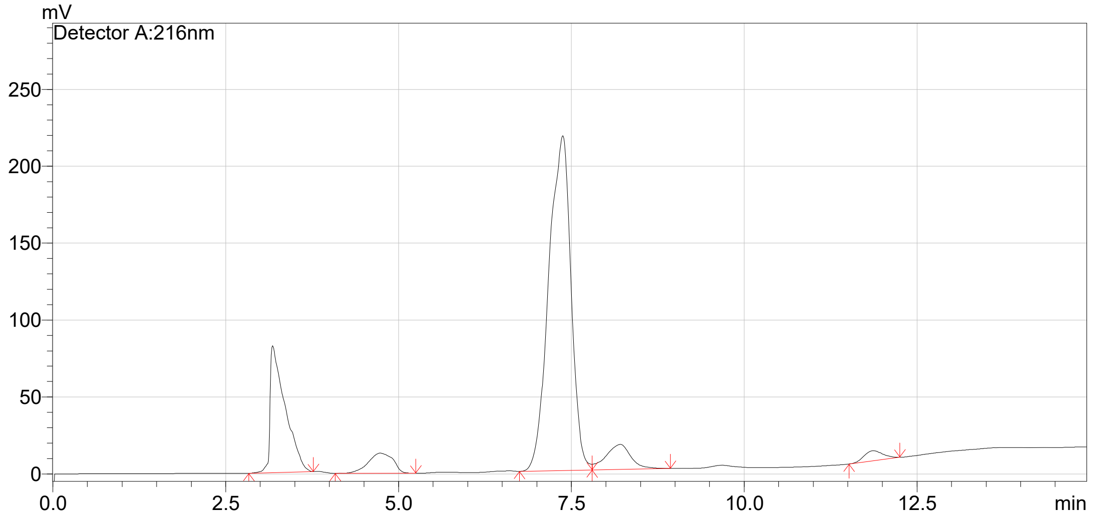

mV
Detector A:216nm
250
200
150
100
50
0
0.0
2.5
5.0
7.5
10.0
12.5
min

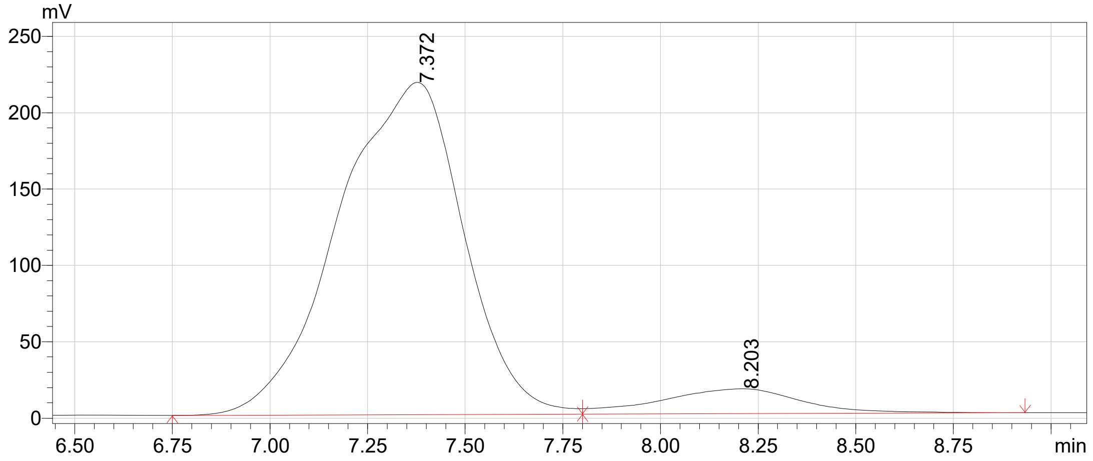

mV
250
200
150
100
50
0
7.372
8.203
6.50
6.75
7.00
7.25
7.50
7.75
8.00
8.25
8.50
8.75
min

4l

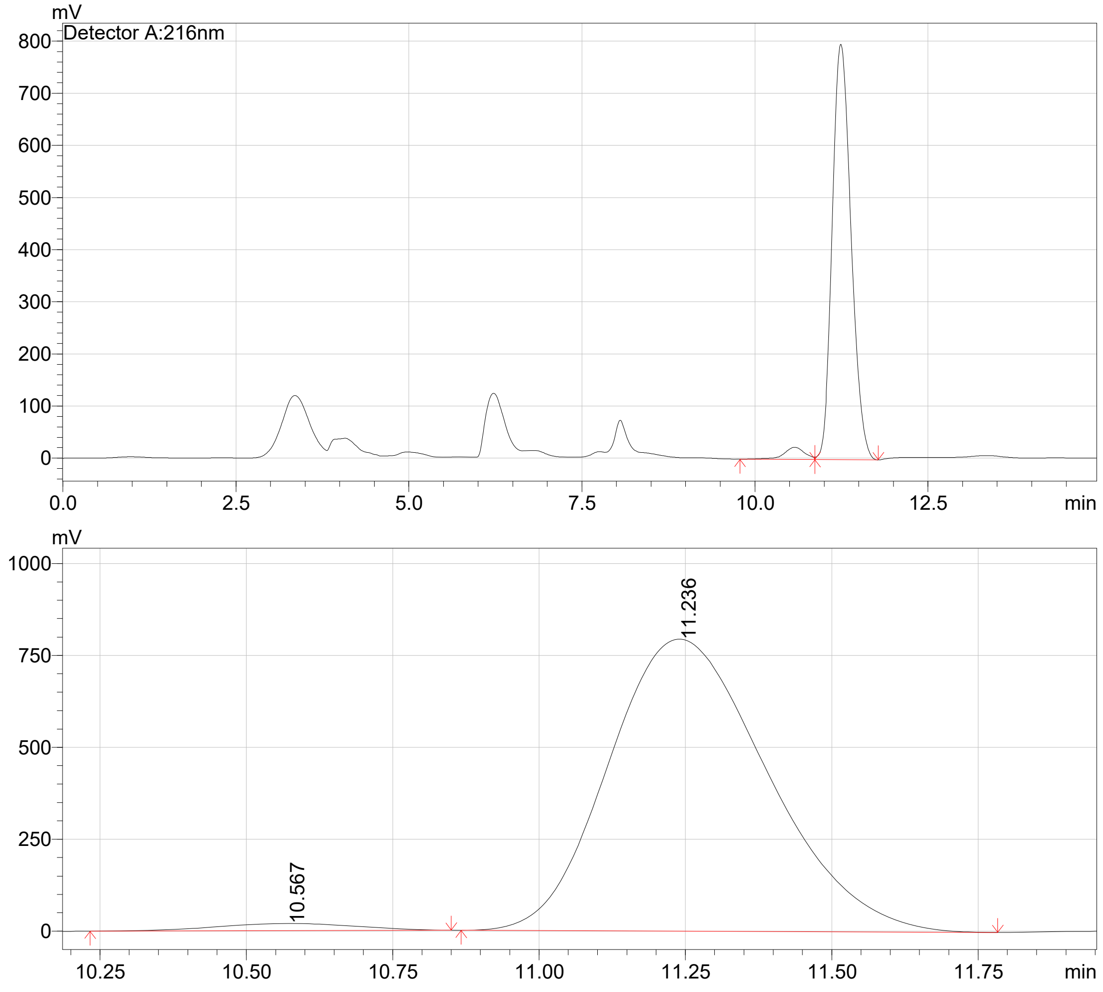

4l (racemate)

Peak Table

Peak#	Ret. Time	Area	Area%	Height	Peak Start	Peak End
1	10.746	3905635	50.2557	262826	10.300	11.167
2	11.493	3865897	49.7443	240529	11.167	12.000

Detector A

Close Help

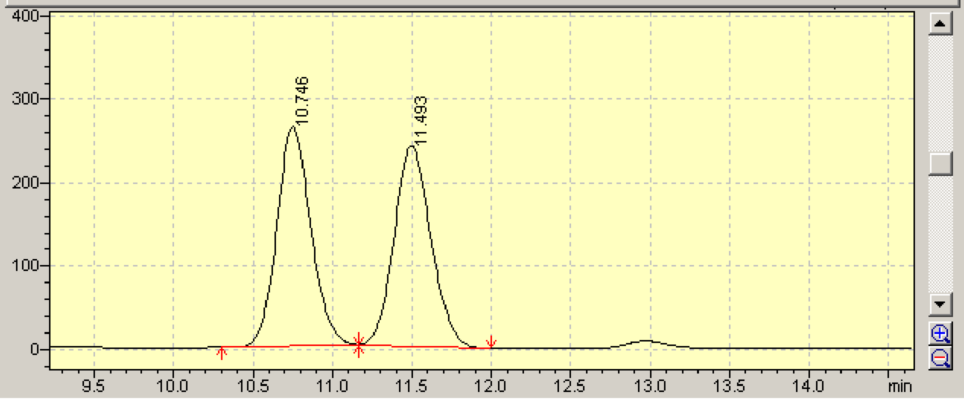

4m

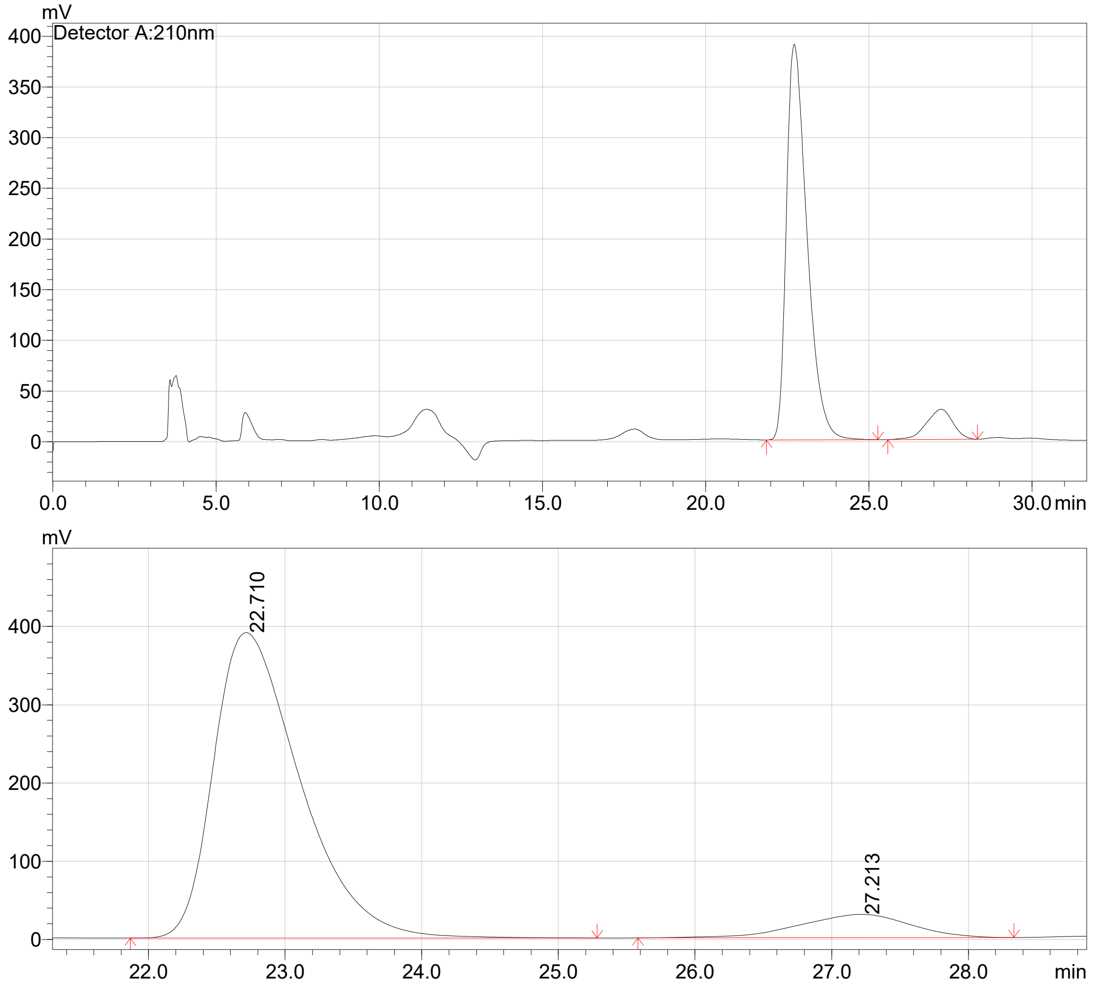

4m (racemate)

Peak Table

Peak#	Ret. Time	Area	Area%	Height	Peak Start	Peak End
1	22.897	9274353	50.3736	235624	22.100	25.267
2	26.649	9136798	49.6264	203025	25.383	27.250

Detector A

Close Help

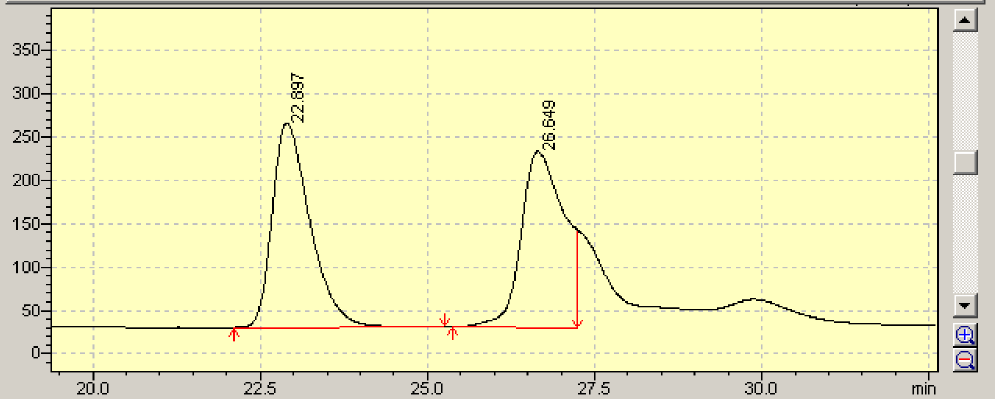

Supplement: Supplementary file 1 [file molecules-27-02538-s001.zip › molecules-1674846-supplementary.pdf]
